# Neuroprotective exendin-4 enhances hypothermia therapy in a model of hypoxic-ischaemic encephalopathy

**DOI:** 10.1093/brain/awy220

**Published:** 2018-08-24

**Authors:** Eridan Rocha-Ferreira, Laura Poupon, Aura Zelco, Anna-Lena Leverin, Syam Nair, Andrea Jonsdotter, Ylva Carlsson, Claire Thornton, Henrik Hagberg, Ahad A Rahim

**Affiliations:** 1 Centre of Perinatal Medicine and Health, Institute of Clinical Sciences, Department of Obstetrics and Gynecology & Institute of Neuroscience and Physiology, Sahlgrenska Academy, University of Gothenburg, Sweden; 2 EGA Institute for Women’s Health, University College London, UK; 3 UCL School of Pharmacy, University College London, UK; 4 Department of Women and Children’s Health, Centre for the Developing Brain, School of Life Course Sciences, King’s College London, UK; 5 Department of Perinatal Imaging and Health, Centre for the Developing Brain, School of Biomedical Engineering and Imaging Sciences, King s College London, UK

**Keywords:** exendin-4, hypoxic-ischaemic encephalopathy, neuroprotection, anti-inflammatory, hypothermia

## Abstract

Hypoxic-ischaemic encephalopathy remains a global health burden. Despite medical advances and treatment with therapeutic hypothermia, over 50% of cooled infants are not protected and still develop lifelong neurodisabilities, including cerebral palsy. Furthermore, hypothermia is not used in preterm cases or low resource settings. Alternatives or adjunct therapies are urgently needed. Exendin-4 is a drug used to treat type 2 diabetes mellitus that has also demonstrated neuroprotective properties, and is currently being tested in clinical trials for Alzheimer’s and Parkinson’s diseases. Therefore, we hypothesized a neuroprotective effect for exendin-4 in neonatal neurodisorders, particularly in the treatment of neonatal hypoxic-ischaemic encephalopathy. Initially, we confirmed that the glucagon like peptide 1 receptor (GLP1R) was expressed in the human neonatal brain and in murine neurons at postnatal Day 7 (human equivalent late preterm) and postnatal Day 10 (term). Using a well characterized mouse model of neonatal hypoxic-ischaemic brain injury, we investigated the potential neuroprotective effect of exendin-4 in both postnatal Day 7 and 10 mice. An optimal exendin-4 treatment dosing regimen was identified, where four high doses (0.5 µg/g) starting at 0 h, then at 12 h, 24 h and 36 h after postnatal Day 7 hypoxic-ischaemic insult resulted in significant brain neuroprotection. Furthermore, neuroprotection was sustained even when treatment using exendin-4 was delayed by 2 h post hypoxic-ischaemic brain injury. This protective effect was observed in various histopathological markers: tissue infarction, cell death, astrogliosis, microglial and endothelial activation. Blood glucose levels were not altered by high dose exendin-4 administration when compared to controls. Exendin-4 administration did not result in adverse organ histopathology (haematoxylin and eosin) or inflammation (CD68). Despite initial reduced weight gain, animals restored weight gain following end of treatment. Overall high dose exendin-4 administration was well tolerated. To mimic the clinical scenario, postnatal Day 10 mice underwent exendin-4 and therapeutic hypothermia treatment, either alone or in combination, and brain tissue loss was assessed after 1 week. Exendin-4 treatment resulted in significant neuroprotection alone, and enhanced the cerebroprotective effect of therapeutic hypothermia. In summary, the safety and tolerance of high dose exendin-4 administrations, combined with its neuroprotective effect alone or in conjunction with clinically relevant hypothermia make the repurposing of exendin-4 for the treatment of neonatal hypoxic-ischaemic encephalopathy particularly promising.

## Teaser

Hypoxic-ischaemic encephalopathy (HIE) causes 25% of neonatal deaths worldwide. Rocha-Ferreira *et al*. demonstrate that a diabetes drug protects the neonatal brain in a model of acute HIE, and confirm that the required receptor is found in human perinatal brain tissue. Synergistic combination with clinical hypothermia enhances therapy, supporting potential translation.

## Introduction

Hypoxic-ischaemic encephalopathy (HIE) is a serious complication of labour caused by reduced blood flow and oxygen supply to the neonatal brain. This can result in mortality for the infant or significant and lasting brain damage. HIE is a global problem with an estimated incidence of 1.5 per 1000 live births. Fifteen to 20% of HIE neonates die during the postnatal period, and an additional 25% develop irreversible and lifelong mental and physical disabilities including cerebral palsy (Shankaran, 2012). In 2010, HIE was associated with 2.4% of the total Global Burden of Disease (Lee *et al.*, 2013).

The known temporal sequence of events in the brain following hypoxia-ischaemia in rodent models has defined a therapeutic window of opportunity of up to ∼8 h based on a time course of secondary energy failure and other measures of secondary brain injury (Blumberg *et al.*, 1997; Gilland *et al.*, 1998*a*, *b*). Early interventions have shown efficacy when initiated early and within this window (Nijboer *et al.*, 2011). Reducing the body temperature (hypothermia) of human neonates within 6 h of hypoxia-ischaemia onset and with duration of 72 h is the only clinically approved treatment (Edwards *et al.*, 2010). Hypothermia is thought to protect neurons by reducing cerebral metabolic rate (Rosomoff and Holaday, 1954) and concentrations of glutamate and nitric oxide (Thoresen *et al.*, 1997), inhibiting cerebral energy failure, preserving high-energy phosphates (Thoresen *et al.*, 1995) and preventing inflammatory cascades (Inamasu *et al.*, 2000). However, while hypothermia therapy is very promising, up to 55% of treated neonates cannot be saved (Gluckman *et al.*, 2005). Therefore, there is a need to develop therapies that are either more effective than hypothermia, or can be used in combination to enhance its therapeutic efficacy.

Exendin-4 (also known as exenatide) is a small peptide drug approved by the Food and Drug Administration (FDA) in 2005 and European Medicines Agency (EMA) in 2006 for the treatment of type 2 diabetes mellitus. It is an analogue of the human glucagon-like peptide-1 (GLP-1) gut hormone peptide that plays a role in regulating blood sugar levels by enhancing insulin production from the pancreas. While GLP-1 has a half-life of ∼1.5 min (Deacon *et al.*, 1996), exendin-4 can reach 60–90 min (Nielsen *et al.*, 2004), making it of therapeutic value, and is administered twice daily after meals. The positive neurological effects of exendin-4 were first recognized by improvements in neuropathic aspects in type 2 diabetes mellitus patients under treatment (Grant *et al.*, 2011). Exendin-4 also efficiently crosses the blood–brain barrier (Kastin and Akerstrom, 2003; Zanotto *et al.*, 2017) and its cellular receptor (GLP1R) is found throughout the brain (Wei and Mojsov, 1995). Although there is no definitive unifying mechanism, *in vitro* and *in vivo* studies have suggested that exendin-4 has neuroprotective, neurotrophic (Perry *et al.*, 2002), neurogenic (Bertilsson *et al.*, 2008), anti-inflammatory (Teramoto *et al.*, 2011), anti-apoptotic (Tews *et al.*, 2009) and mitoprotective (Fan *et al.*, 2010; Kang *et al.*, 2015) properties. These findings, together with the excellent safety profile of exendin-4 in patients with type 2 diabetes mellitus, has led to recent and ongoing clinical trials examining the neuroprotective properties of exendin-4 in patients with Parkinson’s (NCT01174810; NCT01971242; Aviles-Olmos *et al.*, 2013*a*; Athauda *et al.*, 2017) and Alzheimer’s disease (NCT01255163). Recently, the Parkinson’s disease trial has reported that patients on exendin-4 show a statistically significant improvement in clinical motor and cognitive measures compared to control group. This effect persisted 12 weeks after ending the exendin-4 treatment (Athauda *et al.*, 2017).

Given the need to develop effective treatments for neonatal HIE, the encouraging animal and clinical studies supporting the neuroprotective properties of exendin-4 make it an attractive therapeutic option. Little is known regarding the use of exendin-4 in perinatal animals, and there are no neonatal clinical studies. Therefore, we conducted a preclinical assessment of exendin-4 using an established mouse model of neonatal hypoxia-ischaemia causing widespread cerebral damage (Carlsson *et al.*, 2012; Rocha-Ferreira *et al.*, 2015). In this study, we confirmed the presence of GLP1R in the perinatal human and murine brains. We demonstrated significant dose-dependent neuroprotective and anti-inflammatory effect of exendin-4 treatment that is dose-dependent and the effects of which can be maintained even when administration is delayed post-hypoxia-ischaemia. Furthermore, we conducted a toxicity study to examine the safety of high dose repeat administration of exendin-4 in perinatal mice and demonstrate safety and tolerance to the drug. Finally, we established its ability to be used synergistically with therapeutic hypothermia that enhances neuroprotection and ameliorates brain damage.

## Materials and methods

### Study approval

All UK mice experiments were approved by the Ethics Committee of the University College London and were carried out by licensed personnel (PPL PCC436823) in concordance with the UK Home Office Guidelines [Animals (Scientific procedures) Act, 1986]. All mice experiments undertaken in Sweden conformed to the Swedish Board of Agriculture and were approved by the Gothenburg Animal Ethics Committee (61-2014 and 01-2016). CD1 mice (Charles River) were bred in house with a 12-h light/dark cycle and had free access to food and water. Breeding cages contained igloo housing with free access to an exercise plate. Once weekly, enrichment food in the form of dry nuts and fruits was sprinkled onto the cages to allow foraging. All animal experiments included male and female littermates randomly allocated to the different experimental groups to reduce bias and followed the ARRIVE guidelines (Kilkenny *et al.*, 2011). For the human post-mortem preterm study, brain tissue samples were collected from Perinatal Pathology autopsy services at Hammersmith Hospital and St Thomas’s Hospital, London, UK. Parental consent was received in accordance to the guidelines of the National Health Services (NHS), UK. Study ethics were obtained from the Hammersmith and Queen Charlotte’s and Chelsea Research Ethics Committee, NHS Research Ethics Services, UK [Post-mortem magnetic resonance imaging study on developing brain (supported by the Medical Research Council, UK); ethics number 07/H0707/139].

### Blood analysis

Postnatal Day 7 (P7) mice underwent four high doses of exendin-4 (0.5 µg/g per dose, Enzo) via intraperitoneal administration (5 µg/g, 12 h apart). Naïve or saline-injected mice acted as controls (*n* = 6 per group). Twelve hours after the last injection, blood samples taken via cardiac puncture and collected in EDTA-coated tube. The analysis was performed by MRC Harwell Clinical Pathology laboratory (Mary Lyon Centre, UK). Different parameters were obtained: total white blood cells, neutrophils, lymphocytes, monocytes, eosinophils and basophils counts, haematocrit, platelets, red blood cells, haemoglobin and mean corpuscular volume. Blood glucose levels (mmol/l) were measured using a blood glucose monitor (CodeFree, SD Biosensor) in naïve controls and mice following a single exendin-4 high dose administration (0.5 µg/g). Blood samples were collected via cardiac puncture at 0.5 h, 1 h, 2 h, and 4 h post-exendin-4 injection.

### Cyclic AMP assay

Brains of P7 naïve mice were collected at 2 h, 4 h, 8 h and 12 h following a single intraperitoneal injection of exendin-4 (0.5 µg/g). Saline-treated controls were collected 2 h post-injection (*n* = 4 per group). All samples were processed using cAMP direct immunoassay kit as per manufacturer’s instructions (Abcam).

### Hypoxia-ischaemia surgery

Human brain maturation equivalent late preterm (P7) and term (P10) (Semple *et al.*, 2013) CD1 littermate mice were anaesthetized with isoflurane (5% induction, 1.5% maintenance). The left common carotid artery was permanently ligated. Following 1 h recovery, pups were placed in a hypoxic chamber (8% O_2,_ 36°C) for 30 min (P7) or 20 min (P10).

### Hypothermia treatment

Within 10 min post-hypoxia-ischaemia, P10 mice underwent a single high dose of either exendin-4 (0.5 µg/g) or saline and were placed in individual compartments within a hypothermia (33°C) or normothermia (36°C) chamber for 5 h. One probe in each chamber monitored environmental temperature and one animal in each chamber was randomly selected to be used as a temperature-monitoring sentinel to measure core temperature using a rectal probe (T21, 0.41 mm diameter; Physitemp Instruments). The use of a rectal probe is in agreement with previous hypothermia studies in mice and rats demonstrating a good correlation between core body temperature measured this way and brain temperature in small rodent pups (Thoresen *et al.*, 1996*b*). The alternative use of telemetry is promising but more expensive and technically challenging. Room temperature was also recorded. Computer Software Daisy lab 10.0 (Physitemp Instruments) was used to monitor the temperature, and values from each probe were recorded every 5 min (Supplementary Fig. 1). It was decided prior to the experiment that sentinel mice (*n* = 8 normothermia, *n* = 8 hypothermia) were excluded from the final analysis, as restraint-associated stress from the rectal probe has shown a neuroprotective effect (Thoresen *et al.*, 1996*a*).

### Exendin-4 treatment

**Figure 1:**
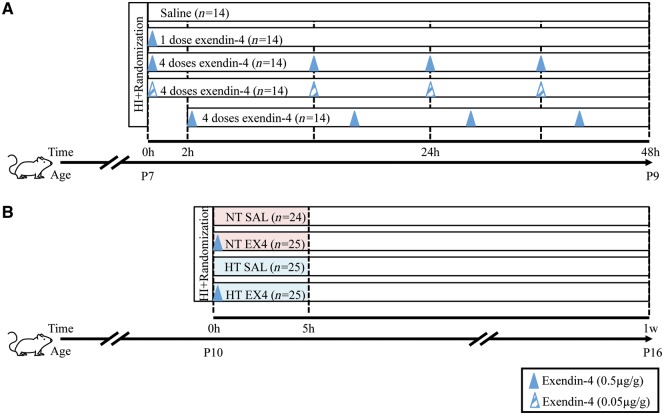
**Schematic of different exendin-4 treatment regimens.** (**A**) Schematic of exendin-4 dosing regimen in the late preterm hypoxia-ischaemia model (P7). (**B**) Schematic of combining clinically relevant hypothermia and exendin-4 treatment in the term hypoxia-ischaemia model (P10). Mouse by Iconic from the Noun Project.

Different dose regimens of exendin-4 were used to establish optimum treatment in the P7 hypoxia-ischaemia injury model, with initiation within the therapeutic window. Animals were randomized to: (i) saline (*n* = 14); (ii) one high dose exendin-4 (0.5 µg/g) administered immediately after hypoxia-ischaemia (*n* = 14); (iii) four high doses of exendin-4 administered every 12 h, starting immediately after hypoxia-ischaemia (*n* = 14); (iv) four low doses of exendin-4 (0.05 µg/g) administered every 12 h, starting immediately after hypoxia-ischaemia (*n* = 14); and (v) four doses of exendin-4 administered every 12 h, starting with a 2 h delay after hypoxia-ischaemia (*n* = 14) (Fig. 1A). In the P10 combined exendin-4 (0.5 µg/g) and hypothermia treatment, animal groups consisted of control normothermia + saline (NT SAL, *n* = 24), single treatments normothermia + exendin-4 (NT EX4, *n* = 25) and hypothermia + saline (HT SAL, *n* = 25), and combined treatment hypothermia + exendin-4 (HT EX4, *n* = 25) (Fig. 1B).

### Tissue preparation

Highest level of widespread neuronal caspase-3 expression within the acute injury phase occurs 48 h post-hypoxia-ischaemia (Johnston *et al.*, 2011). Therefore, this time point was chosen for early evaluation of neuropathological markers in the P7 study. P7 mice were perfused with 4% paraformaldehyde (PFA) 48 h post-hypoxia-ischaemia, brains transferred to 30% sucrose and snap frozen. P10 mice were perfused in the same manner 7 days after hypoxia-ischaemia, a time point when the secondary phase of brain injury is completed (Gilland *et al.*, 1998*a*). Coronal brain sections (40-μm thickness) starting from the point of fusion of the corpus callosum were collected onto TBSAF wells. Every 10th section, in a total of five sections (400 µm apart) per brain underwent histochemistry, immunohistochemistry or immunofluorescence. Whole post-mortem preterm neonatal brains were fixed in 4% formalin for 7 weeks before anatomical positions from the frontal, occipital, and parietal lobes were selected and processed using a Leica tissue processor. The blocks were sectioned at 6-µm thickness using a microtome, mounted onto SuperFrost™ Plus slides and allowed to dry for 4 days.

### Histochemistry

P7 naïve, saline- and high dose exendin-4-treated mice (no hypoxia-ischaemia, *n* = 6 per group) were culled 12 h after the last exendin-4 injection (four doses 12 h apart). Different organs: brain, heart, spleen, liver, lung, pancreas and kidney were stained with haematoxylin and eosin for histopathological assessment.

### Immunohistochemistry

Brain sections were treated as previously described (Rocha-Ferreira *et al.*, 2015). In brief, slides were fixed in 4% PFA, blocked with 5% goat serum (Sigma-Aldrich) for 30 min and incubated overnight with MAP2 (1:1000, Sigma), alphaM (1:5000, Serotec), CD68 (1:2000, Biorad), GFAP (1:6000, Dako) or ICAM1 (1:3000, Pharmingen) antibodies. Sections were then incubated with appropriate biotinylated secondary antibody (anti-rat or anti-rabbit; 1:100) for 1 h at room temperature, followed by 1 h incubation with ABC (1:100; Vector) solution and visualized with 3,3′ diaminobenzidene (DAB)/hydrogen peroxide (Sigma). The reaction was stopped and washed twice in bidistilled water. Slides were dehydrated by consecutive immersion in increasing concentrations of ethanol, isopropanol and xylene, then covered using DEPEX. Post-mortem neonatal sections were stained as previously shown (Carlsson *et al.*, 2011). Slides underwent routine deparaffinization, followed by 15 min 1% hydrogen peroxide/PBS-Tween incubation before 15 min block with 5% goat serum. Overnight incubation with GLP1R (1:100, Novus Bio) was followed by 2 h room temperature incubation with biotinylated goat anti-rabbit secondary antibody (1:1000). Sections were incubated for 1 h with ABC (1:20) and visualized with DAB/hydrogen peroxide. The reaction was stopped, and sections covered as described above.

### TUNEL staining

Murine brain sections were incubated in 3% hydrogen peroxide/methanol (15 min) followed by 2 h incubation at 37°C with terminal deoxytransferase and deoxyuridine triphosphate solution (Roche). The reaction was stopped by incubation in terminal deoxynucleotidyl transferase dUTP nick-end labelling (TUNEL) stop solution for 10 min. Slides were incubated in ABC solution (1 h, room temperature) and visualized using DAB enhanced with cobalt nickel in the presence of hydrogen peroxide. The reaction was stopped, and sections covered as described above.

### Immunofluorescence and scanning confocal microscopy

**Figure 2:**
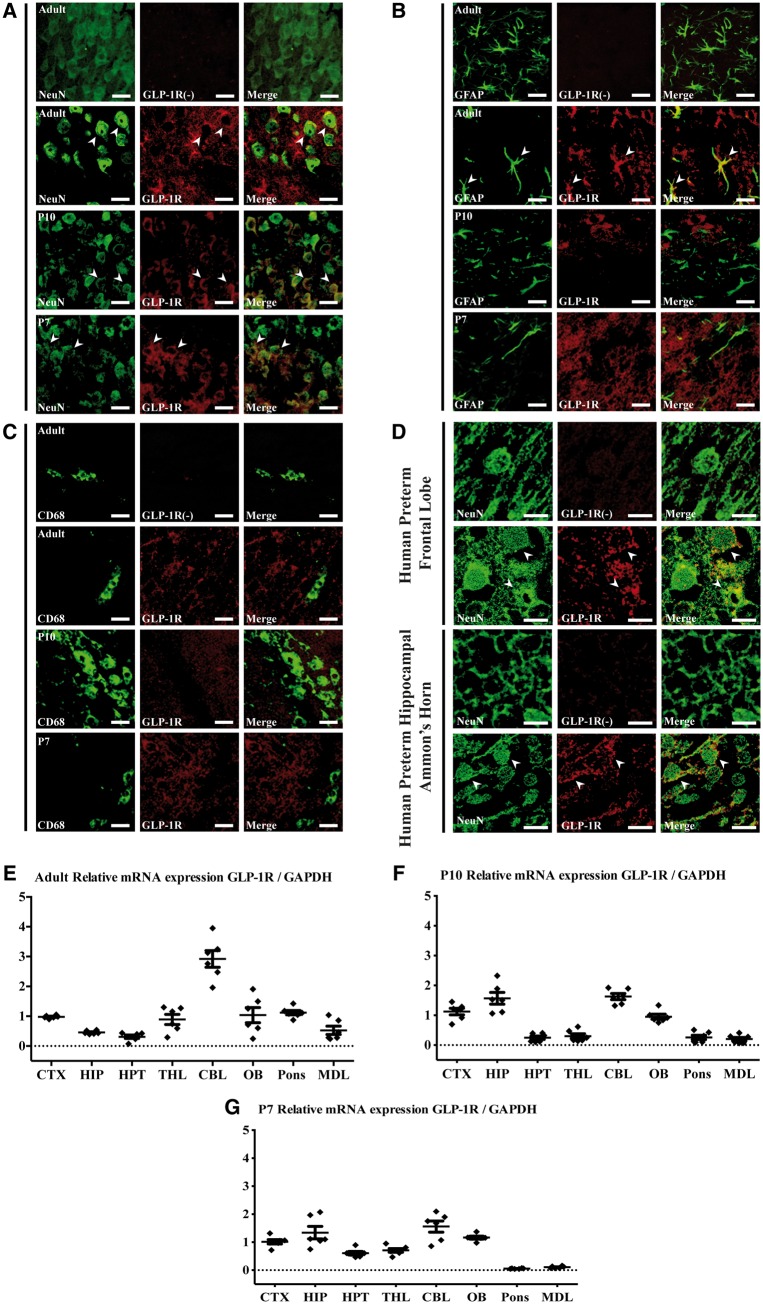
**GLP1R expression in the human preterm and murine brain across different developmental ages.** (**A**) Immunofluorecence and scanning confocal microscopy studies demonstrating GLP1R is expressed in neurons (NeuN) in the mouse brain at 10 weeks (adult), P10 and P7, (**B**) with only small colocalization with astrocytes (GFAP) at 10 weeks of age, and (**C**) no co-localization with microglia (CD68) cells in any of the different developmental ages. The first *row* of micrographs (**A**–**C**) show negative staining for GLP1R antibody. (**D**) Immunofluorescence studies of post-mortem human preterm brain tissue shows co-localization of GLP1R expression in neurons (NeuN) in the frontal lobe and hippocampus Ammon’s horn, with the first row of micrographs for each human brain region containing negative staining for GLP1R antibody. A median filter was applied to the images to reduce noise. (**E**) Relative quantification of GLP1R expression by quantitative PCR in different brain regions at 10 weeks, (**F**) P10 and (**G**) P7 (*n* = 6 per age group). CBL = cerebellum; CTX = cortex; HIP = hippocampus; HPT = hypothalamus; MDL = medulla; OB = olfactory bulbs; THL = thalamus. Scale bar = 20 µm in **C**; 10 µm in **D**.

Naïve P7, P10 and adult 10 week CD1 mice brain sections (*n* = 3 per group) were stained by simultaneous overnight incubation with rabbit anti-GLP1R (1:50, Novus Bio) and mouse anti-NeuN (1:1000, Millipore), mouse anti-GFAP (1:2000, Millipore) or rat anti-CD68 (1:2000, Bio-Rad) antibodies. Representative images were captured using a Zeiss LSM710 confocal microscope and Zen software (Carl Zeiss AG). Post-mortem neonatal sections were simultaneously incubated with GLP1R (1:100) and NeuN (1:500) antibodies. Sections were treated with fluorochrome-conjugated secondary antibody (1:1000, Alexa Fluor^®^ 488 and 546) and mounted with a Fluoromount-G^™^ mounting medium (ThermoFisher Scientific). Representative images were captured using a Leica SP5 confocal microscope equipped with LAS-AF software. All images were processed using Imaris v.9.1 (Bitplane AG). Because of known difficulties associated with the GLP1R antibodies (Drucker, 2013), negative GLP1R antibody stained sections were also used for all developmental time points and in both murine and human tissue (Fig. 2 and Supplementary Fig. 2).

### Quantitative polymerase chain reaction

The distribution of GLP1R gene expression was assessed in different brain regions: olfactory bulb, cortex, hypothalamus, thalamus, hippocampus, cerebellum, pons and medulla. Naïve brains were extracted at P7, P10 and 10 weeks, and RNA was isolated using RNeasy® Mini kit (Qiagen) and first-strand cDNA was generated using High-Capacity cDNA Reverse Transcription kit (Applied Biosystems). Quantitative RT-PCR was carried out using a StepOnePlus (Applied Biosystems) with the SsoAdvanced™ Universal SYBR® Green PCR Core Reagents Supermix (Bio-Rad). Primers were designed to detect mouse *Glp1r*: forward 5′-AGACGGTGCAGAAATGGAGA-3′; reverse 5′-TGGCGCTTCCGTGAGG-3′ and *Gapdh* housekeeping gene: forward 5′-GTTGTCTCCTGCGACTTCA-3′; reverse 5′- GGTGGTCCAGGGTTTCTTA-3′. Data from StepOne™ software v2.3 were calibrated to *Gapdh* and the relative quantitation of gene expression was performed using the comparative CT method.

### Data analysis

Infarct area was measured using ImageJ software (NIH). The intact Nissl+ areas of the isocortex, pyriform cortex, hippocampus, striatum, thalamus and external capsule brain regions were delineated bilaterally and the ipsilateral regions were subtracted from the contralateral regions and expressed as percentage tissue loss. This method assumes that the contralateral hemisphere represents maximal intact area (100%); however, there are instances where because of potential symmetrical differences between hemispheres, the intact ipsilateral hemisphere is larger in area size and the resulting measurement shows a negative value. TUNEL+ cells were quantified under ×20 magnification in three separate fields per assessed brain region. Microglial activation was assessed using semi-quantitative score as previously described (Rocha-Ferreira *et al.*, 2015), with a scale of: 0 (no activation, ramified microglia) to 4 (widespread amoeboid microglia). GFAP, ICAM1 and CD68 immunoreactivity was measured by quantitative thresholding image analysis using Image Pro Premier software (Media Cybernetics) as previously described (Rocha-Ferreira *et al.*, 2016). In brief, three non-overlapping RGB images from the assessed brain regions were captured using a live video camera (Nikon, DS-Fi1) mounted onto a Nikon eclipse E600 microscope at ×40 magnification for both GFAP and ICAM1 stainings. Similarly, 30 non-overlapping RBG images from brain, heart, spleen, liver, lung, pancreas and kidney were captured for CD68 measurements. The threshold setting was kept constant for all acquired images. MAP-2 staining was used for quantification of volumetric tissue loss from five brain levels, starting from level 1 (L1, fusion of corpus callosum) and continuing until level 5 (L5, late hippocampus), as well as total volumetric tissue loss (Stridh *et al.*, 2013). Macroscopic score of tissue loss was performed using a scale comprising of 0, no visible injury; 1, 25%; 2, 50%; and 3, 75% hemispheric injury/loss.

### Statistical analysis

Experimental cohorts consisted of 14–25 mice, based on power calculations as described by Dupont and Plummer (1990). The levels of significance are 5% with 80% power as a minimum. The noise and effect size were estimated through our previous experience and publications using this mouse model (Wang *et al.*, 2010; Carlsson *et al.*, 2011) and calculations were performed using publicly available software, PS: Power and Sample Size Calculation v3.1.2, 2014 (http://biostat.mc.vanderbilt.edu/wiki/Main/PowerSampleSize) (Dupont and Plummer, 1998). Data were analysed using the GraphPad Prism v6.0. All assessments were performed blindly. Average ± standard error of the mean (SEM) was recorded for all data (Supplementary Table 1) and was first analysed with the Kolmogorov–Smirnov normality test. As the data did not follow Gaussian distribution, the Kruskal–Wallis non-parametric test was applied, followed by Dunn’s test (Supplementary Table 2). *P < 0.05, **P < 0.01, ***P < 0.001 and ****P < 0.0001.

### Data availability

All raw data are available from the corresponding author on request.

## Results

### GLP1R is expressed in the human and murine neonatal brain

Firstly, we sought to confirm the presence of GLP1R both in the human and murine brains. Confocal imaging using antibodies against GLP1R and neural cell-specific markers showed GLP1R co-localization predominantly in neurons (NeuN) in all assessed developmental ages: adult (10 weeks), P10 and P7 (Fig. 2A). At 10 weeks of age, co-localization of GLP1R was observed mostly in neurons but also in astrocytes (GFAP). This astrocyte co-localization was not observed at P10 and P7 (Fig. 2B). No microglia (CD68) co-localization with GLP1R was observed in any of the different developmental ages (Fig. 2C). Post-mortem neonatal sections showed GLP1R expression in the frontal lobe and hippocampus Ammon’s horn. Confocal imaging confirmed specific co-localization with neurons (NeuN) (Fig. 2D). Quantitative PCR analysis using primers against *Glp1r* and control *Gapdh* mRNA for normalization of data (Supplementary Fig. 2) revealed GLP1R expression across the different brain regions examined in adult (10 weeks) and neonatal (P10 and P7) mice. *Glp1r* mRNA was particularly present in the cortex, cerebellum and olfactory bulb of adult (Fig. 2E), P10 (Fig. 2F) and P7 mice (Fig. 2G), and also in the hippocampus of P10 and P7 mice.

### Exendin-4 reduces brain infarction in a dose- and time-dependent manner

**Figure 3:**
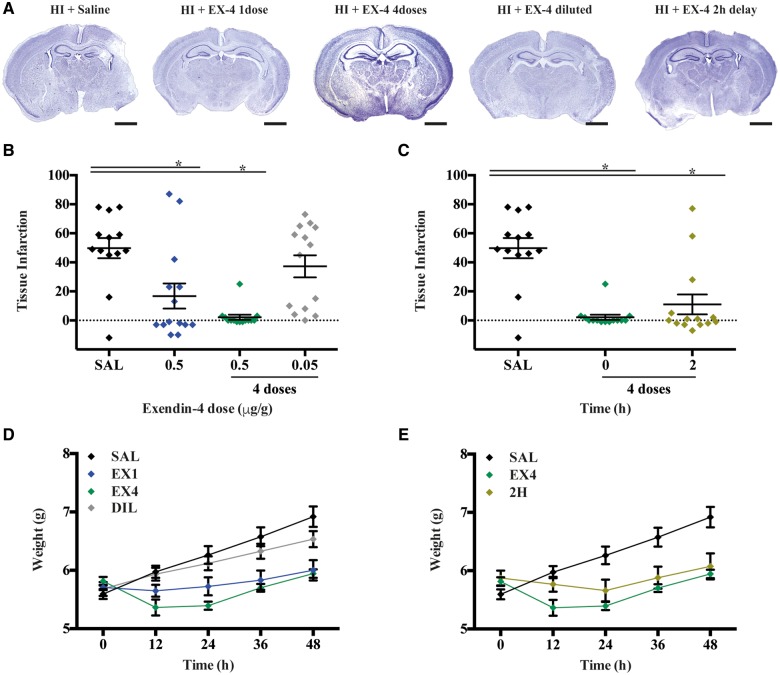
**Evaluation of optimal exendin-4 dose and time treatment regimen in the P7 late preterm model.** (**A**) Representative whole brain micrographs of the different treatment groups: saline (*n* = 14, eight male and six females); one high dose exendin-4 (*n* = 14, eight male and six female), four high doses exendin-4 (*n* = 14, seven male and seven female) and four low-doses exendin-4 (*n* = 14, seven male and seven female) started immediately after hypoxia-ischaemia, and four high doses exendin-4 initiated 2 h after hypoxia-ischaemia (*n* = 14, seven male and seven female). (**B**) Effect on ipsilateral hemispheric tissue loss of different dose regimen started immediately after hypoxia-ischaemia and (**C**) delayed start of exendin-4 treatment. (**D**) Weight gain over a 48 h period following immediate exendin-4 administration at the different doses and (**E**) different time. Data presented as individual animals ± SEM and analysed using Kruskal-Wallis Dunn’s test. *P < 0.05, **P < 0.01, ***P < 0.001. ****P < 0.0001. Scale bar = 2 mm. EX-4 = exendin-4; HI = hypoxia-ischaemia.

To assess the efficacy of exendin-4 brain protection against neonatal hypoxia-ischaemia, tissue infarction was measured 48 h post-insult using different dosing and concentration regimen, and timing of intervention. Saline-treated hypoxia-ischaemia littermates served as controls (Fig. 3A). The Nissl measurement as a percentage of tissue loss showed overall profound and consistent injury in the ipsilateral hemisphere of saline-treated hypoxia-ischaemia controls (SAL, 50% ± 6.9%). A single high dose intraperitoneal administration of exendin-4 immediately after hypoxia-ischaemia significantly reduced tissue infarction (17% ± 8.6%, *P* = 0.0279). The same high dose given immediately after hypoxia-ischaemia and then every 12 h over a 48-h period provided added reduction of brain injury (2% ± 1.8%, *P* = 0.0214). However, dilution of exendin-4 (0.05 µg/g) given in the same four-dose regimen was not protective (37% ± 8%) (Fig. 3B). Delaying the first administration of exendin-4 by 2 h, in the same 12-h apart four high dose administrations still resulted in a significant protective effect (11.0% ± 6.9%, *P* = 0.0334), with no difference in protection observed between immediate and delayed four high dose regimens (Fig. 3C). Breakdown of Nissl measurements across individually assessed brain regions showed that immediate start of four high dose exendin-4 administrations had the highest regional protection: isocortex (*P* = 0.0222), pyriform cortex (*P* = 0.0629), hippocampus (*P* = 0.0005) and striatum (*P* = 0.0064). This is followed by the same four high dose administrations started 2 h after hypoxia-ischaemia: isocortex (*P* = 0.0334), striatum (*P* = 0.0069) and thalamus (*P* = 0.0406). Lastly, single high dose exendin-4 given immediately after hypoxia-ischaemia also reduced regional brain injury in pyriform cortex (*P* = 0.0013) and striatum (*P* = 0.0468) (Supplementary Fig. 3). There were no differences between the different hypoxia-ischaemia-saline control groups, therefore, animals from all saline groups were randomly pooled. There were no differences between males and females in response to any of the treatments and the distribution of the sexes of mice per experiment are listed in Supplementary Table 3.

Weight gain measurement was performed in all groups. Following hypoxia-ischaemia, a single high dose administration of exendin-4 did not cause weight loss; however, there was a reduced weight gain in comparison to saline controls, which reached significance at the 36 h (*P* = 0.0212) and 48 h (*P* = 0.0148) time points post-hypoxia-ischaemia. Both immediate and 2-h delay four high dose exendin-4 treatments resulted in initial non-significant weight loss when compared to baseline weight, with visible signs of recovery 48 h post-hypoxia-ischaemia. In the exendin-4 (four doses group) mice weighed significantly less than the low-dose group at 12 h (*P* = 0.0150) and 24 h (*P* = 0.0096) time points, and significantly less than saline-injected mice at 24 h (*P* = 0.0074), 36 h (*P* = 0.0315) and 48 h (*P* = 0.0269) post-hypoxia-ischaemia (Fig. 3D). There was no exendin-4-mediated modulation of weight in the 2-h-delayed four-dose treatment (2H) and low-dose-treated groups. To assess how quickly exendin-4 was crossing the blood–brain barrier and potentially initiating a cellular response in the CNS, we measured alterations in cyclic AMP (cAMP) expression, a known second messenger of GLP1R signalling. Naïve animals were given one high dose of exendin-4 and cAMP was measured in the brain at 2 h, 4 h, 8 h and 12 h. Significantly higher total brain cAMP was measured at 2 h (*P* = 0.0021) and 4 h (*P* = 0.0281) when compared to saline-injected controls at 2 h (Supplementary Fig. 4).

### Exendin-4 prevents cell death and neuroinflammation

**Figure 4:**
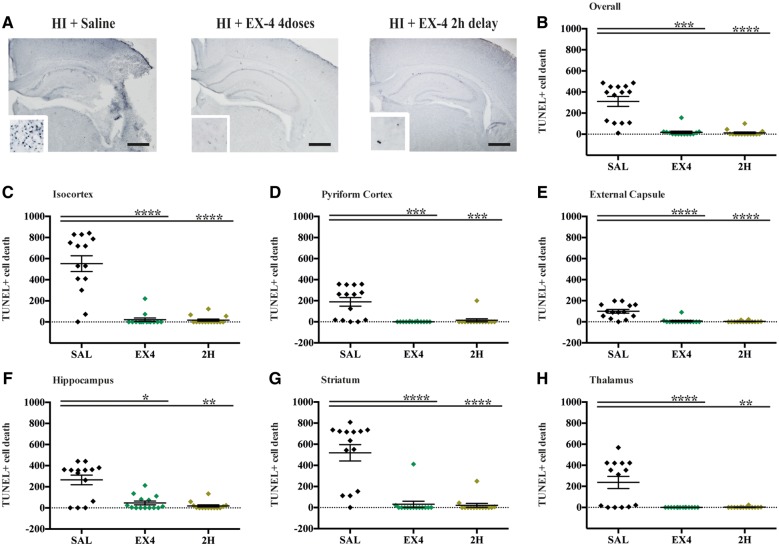
**Exendin-4 treatment reduces cell death 48 h after P7 hypoxia-ischaemia.** (**A**) Hippocampus micrograph representation with high magnification inserts (×40), of saline, immediate and 2 h delayed four high dose exendin-4 treatment. (**B**) Overall and individual (**C**) isocortex, (**D**) pyriform cortex, (**E**) external capsule, (**F**) hippocampus, (**G**) striatum and (**H**) thalamus brain regions quantification of TUNEL+ cell death 48 h after hypoxic-ischaemic injury across the three different treatment groups. Data presented as number of TUNEL+ cells per individual animal ± SEM and analysed using Kruskal-Wallis Dunn’s test. *P < 0.05, **P < 0.01, ***P < 0.001 and ****P < 0.0001. Scale bar = 400 µm. HI = hypoxia-ischaemia.

**Figure 5:**
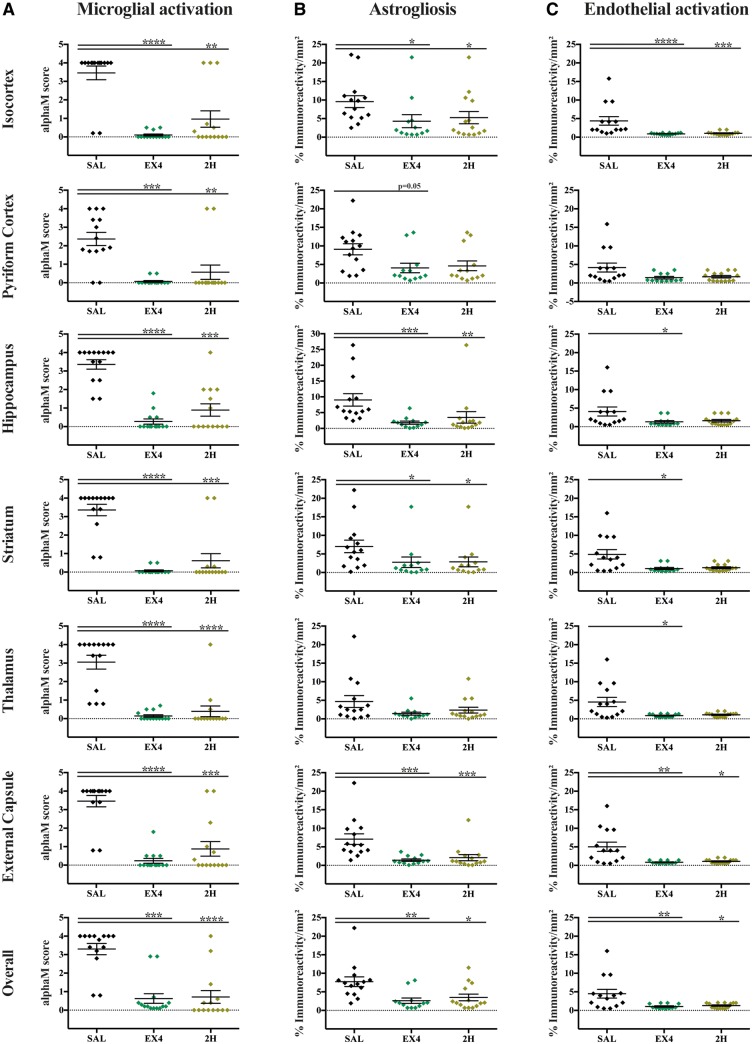
**Exendin-4 suppresses glial cell activation 48 h after hypoxia-ischaemia.** Overall and regional assessment of microglia activation (alphaM, αmβ2) through semi-quantitative score (**A**), and quantitative immunoreactivity analysis of astrocytes (GFAP) (**B**) and endothelial cells (ICAM1) (**C**). Data presented as individual animals ± SEM and analysed using Kruskal-Wallis Dunn’s test. *P < 0.05, **P < 0.01, ***P < 0.001 and ****P < 0.0001.

Evaluation of cell death as quantification of TUNEL+ cells 48 h after late preterm hypoxia-ischaemia demonstrated substantial overall cell loss in the control saline-treated group (310.5 ± 62.9) (Fig. 4A). Four administrations of high dose exendin-4 started either immediately post-hypoxia-ischaemia or 2 h later significantly reduced the overall number of TUNEL+ cells (*P* = 0.0002 and *P* < 0.0001, respectively) (Fig. 4B). Furthermore, this protective effect was significant across all individually assessed brain regions: isocortex (SAL versus EX4, *P* < 0.0001, SAL versus 2H, *P* < 0.0001) (Fig. 4C), pyriform cortex (SAL versus EX4, *P* = 0.0001, SAL versus 2H, *P* = 0.0002) (Fig. 4D), external capsule (SAL versus EX4, *P* < 0.0001, SAL versus 2H, *P* < 0.0001) (Fig. 4E), hippocampus (SAL versus EX4, *P* = 0.0253, SAL versus 2H, *P* = 0.0037) (Fig. 4F), striatum (SAL versus EX4, *P* < 0.0001, SAL versus 2H, *P* < 0.0001) (Fig. 4G) and thalamus (SAL versus EX4, *P* < 0.0001, SAL versus 2H, *P* = 0.0010) (Fig. 4H). Microglial activation (alphaM, αmβ2) was significantly reduced in both the four administrations of exendin-4 (*P* = 0.0006) and 2 h delayed intervention cohorts (*P* < 0.0001) compared to the saline-treated controls (Fig. 5A and Supplementary Fig. 5). This effect was observed throughout all assessed brain regions: isocortex (SAL versus EX4, *P* < 0.0001, SAL versus 2H, *P* = 0.0024), pyriform cortex (SAL versus EX4, *P* = 0.0002, SAL versus 2H, *P* = 0.0011), hippocampus (SAL versus EX4, *P* < 0.0001, SAL versus 2H, *P* = 0.0005), striatum (SAL versus EX4, *P* < 0.0001, SAL versus 2H, *P* = 0.0001), thalamus (SAL versus EX4, *P* < 0.0001, SAL versus 2H, *P* < 00001) and external capsule (SAL versus EX4, *P* < 0.0001, SAL versus 2H, *P* = 0.0006). Astrogliosis (GFAP) was substantially suppressed in both the four administrations of exendin-4 (*P* = 0.0038) and 2-h delayed intervention cohorts (*P* = 0.0155) compared to the saline-treated controls (Fig. 5B and Supplementary Fig. 5). Regional analysis showed both immediate and 2-h delayed four-dose regimen treatments had significantly less GFAP in the isocortex (*P* = 0.0115 and *P* = 0.0416), hippocampus (*P* = 0.0006 and *P* = 0.0011), striatum (*P* = 0.0279 and *P* = 0.0412) and external capsule (*P* = 0.0004 and *P* = 0.0005, respectively). The pyriform cortex of EX4 mice had significantly less astrogliosis (*P* = 0.0551) than SAL-treated hypoxic-ischaemic mice. Immediate administration of the four doses of exendin-4 showed the highest inhibition of endothelial activation (ICAM1) (*P* = 0.0050) (Fig. 5C and Supplementary Fig. 5). This effect was present in most assessed brain regions: isocortex (*P* < 0.0001), hippocampus (*P* = 0.0443), striatum (*P* = 0.0136), thalamus (*P* = 0.0171) and external capsule (*P* = 0.0057). Four doses of exendin-4 administration with a 2-h delay inhibited the overall increase in ICAM1 (*P* = 0.0356), which was also observed in the isocortex (*P* = 0.0004) and external capsule (*P* = 0.0300).

### Repeated high doses of exendin-4 is non-toxic in neonatal mice

**Figure 6:**
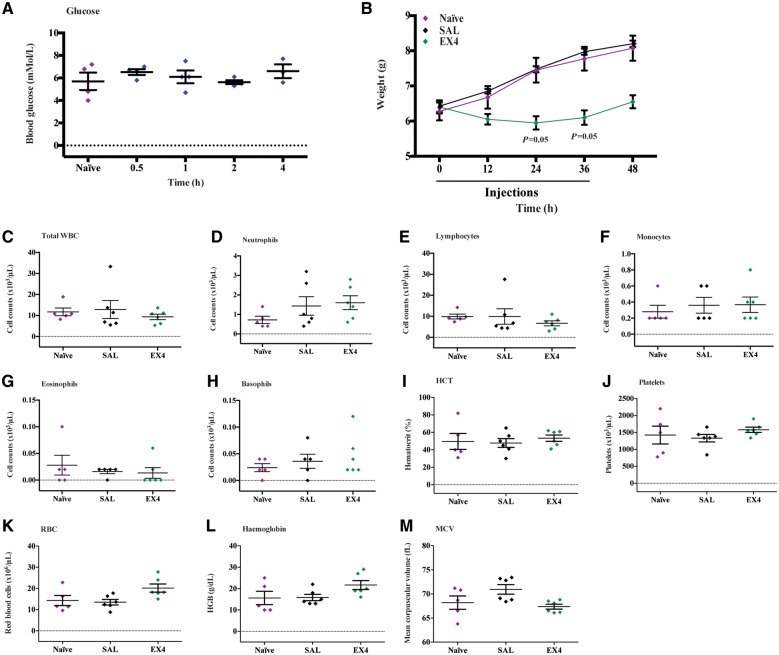
**Exendin-4 administration blood analysis.** (**A**) Blood glucose measurement at different time points in naïve and treated mice following high dose (0.5 µg/g) exendin-4 injection (*n* = 4 per group). (**B**) Weight measurement in naïve, saline and exendin-4 alone during the four high dose administration regimen. (**C**) Total white blood cell (WBC), (**D**) neutrophils, (**E**) lymphocytes, (**F**) monocytes, (**G**) eosinophils and (**H**) basophils counts, (**I**) haematocrit (HCT), (**J**) platelets, (**K**) red blood cells (RBC), (**L**) haemoglobin and (**M**) mean corpuscular volume (MCV) at the end of the treatment regimen. Data presented as individual animals or as mean ± SEM and analysed using Kruskal-Wallis Dunn’s test. *P < 0.05, **P < 0.01, ***P < 0.001 and ****P < 0.0001. EX4 = exendin-4; SAL = saline.

**Figure 7:**
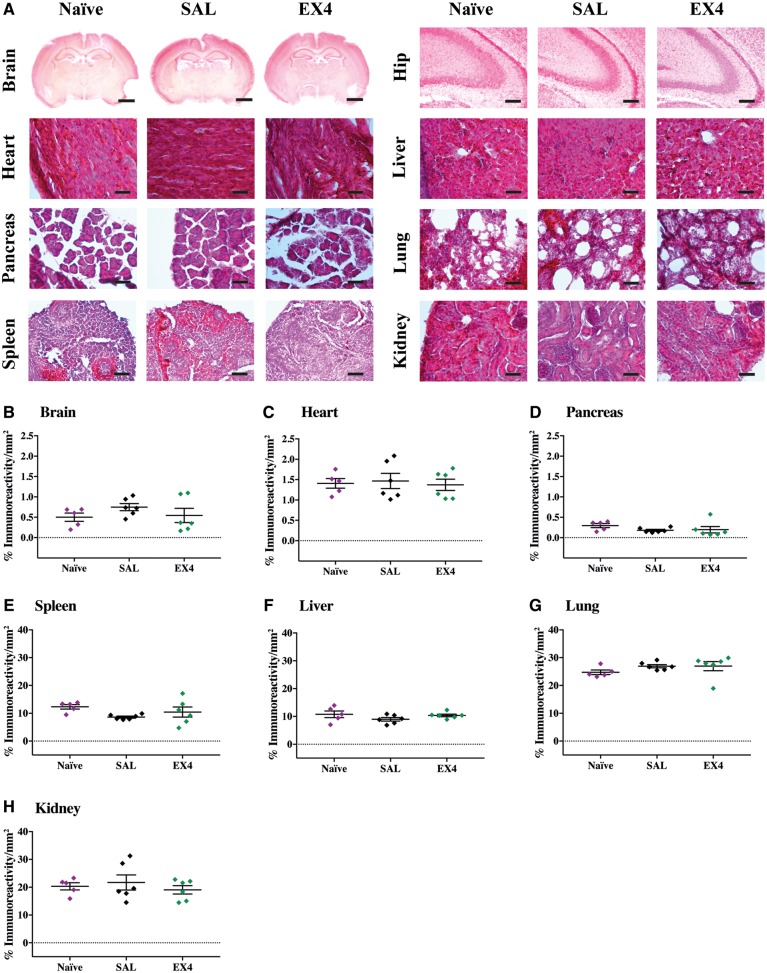
**Histopathological organ assessment.** (**A**) Representative micrographs of haematoxylin and eosin stained brain, including hippocampus (hip) level and visceral organs: heart, liver, pancreas, spleen, lung and kidney showing no abnormal histopathology in naïve, saline (SAL) or exendin-4 (EX4) alone treatments. (**B**) Quantitative immunoreactivity threshold measurements of macrophages (CD68) in the brain, (**C**) heart, (**D**) spleen, (**E**) liver, (**F**) lung, (**G**) pancreas and (**H**) kidney for the different groups (*n* = 6 per group). Data presented as individual animals ± SEM and analysed using Kruskal-Wallis Dunn’s test. *P < 0.05, **P < 0.01, ***P < 0.001 and ****P < 0.0001.

The therapeutically optimal exendin-4 dose (0.5 µg/g) given every 12 h over a 48-h period in this study are substantially higher than those used clinically in diabetic patients. Therefore, we conducted an examination in naïve (no hypoxia-ischaemia) P7 mice for any toxicity or adverse effects. High dose exendin-4 did not alter blood glucose levels when compared to saline-treated or naïve controls (0 h, 0.5 h, 1 h, 2 h and 4 h time points) (Fig. 6A). The exendin-4 four high dose regimen in naïve animals resulted in reduced weight gain at 24 h (naïve versus EX4, *P* = 0.0552, SAL versus EX4, *P* = 0.0552) and 36 h (naïve versus EX4, *P* = 0.0558, SAL versus EX4, *P* = 0.05598 time points) (third and fourth injections), with partial recovery to baseline weight measurement (0 h time point) by 48 h after final injection (Fig. 6B). As exendin-4 has been shown to modulate peripheral immune cells (Yanay *et al.*, 2015; He *et al.*, 2017), blood tests were conducted after completion of the four high dosing regimen. Counts of various blood cell populations and biochemistry: total white blood cells (Fig. 6C), neutrophils (Fig. 6D), lymphocytes (Fig. 6E), monocytes (Fig. 6F), eosinophils (Fig. 6G), basophils (Fig. 6H), haematocrit (Fig. 6I), platelets (Fig. 6J), red blood cells (Fig. 6K), haemoglobin (Fig. 6L) and mean corpuscular volume (Fig. 6M) in mice treated with the high dose exendin-4 regimen were all normal with no significant differences to naïve unadministered mice and saline-injected mice. Given the systemic administration of exendin-4, in addition to the brain we also harvested the heart, spleen, liver, lung, pancreas and kidney for analysis. Haematoxylin and eosin staining did not reveal any obvious fibrosis or abnormalities in cellular or tissue architecture in mice receiving the high dose exendin-4 regimen when compared to naïve or saline-treated mice (Fig. 7A). Quantitative threshold image analysis using a macrophage-specific marker (CD68) did not show any significant inflammatory response in any of the organs examined from the exendin-4-administered mice compared to controls (Fig. 7B–H and Supplementary Fig. 5).

### High dose exendin-4 enhances hypothermia neuroprotection

**Figure 8:**
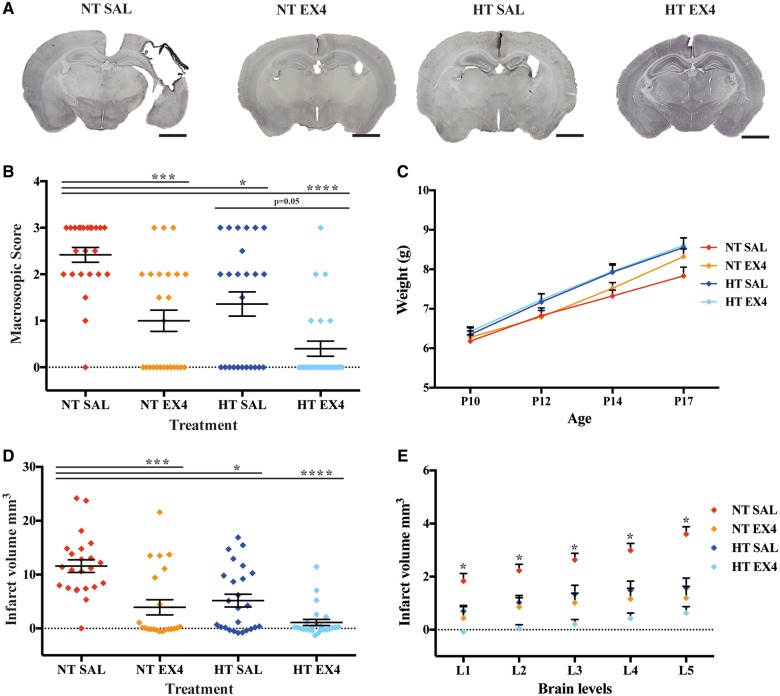
**Synergistic enhanced neuroprotection following combined exendin-4 and hypothermia treatment in the term hypoxia-ischaemia model.** (**A**) Whole brain representative micrographs of normothermia + saline control (NT SAL, *n* = 24, 10 male and 14 female), single treatments normothermia + exendin-4 (NT EX4, *n* = 25, 12 male and 13 female) and hypothermia + saline (HT SAL, *n* = 25, 14 male and 11 female), as well as combined hypothermia and exendin-4 treatment (HT EX4, *n* = 25, 12 male and 13 female) 7 days after P10 hypoxic-ischaemic injury. (**B**) Macroscopic score evaluation. (**C**) Weight gain across the different groups. (**D**) Overall infarct volume and (**E**) through different injury levels, where level 1 indicates the most anterior level, as assessed using MAP-2-stained sections from the different treatments. Data presented as individual animals or as mean ± SEM and analysed using Kruskal-Wallis Dunn’s test. *P < 0.05, **P < 0.01, ***P < 0.001 and ****P < 0.0001. Scale bar = 2 mm.

Following hypoxia-ischaemia, animals were randomly allocated into four groups: (i) normothermia and a single saline injection; (ii) normothermia and a single high dose exendin-4; (iii) hypothermia and a single saline injection; and (iv) hypothermia and a single high dose exendin-4 (Fig. 8A). The sex of each animal is shown in Supplementary Table 3. Macroscopic visual scoring of brain injury 7 days post-hypoxia-ischaemia demonstrated a more pronounced loss of tissue in the normothermia + saline (NT SAL) control group when compared to normothermia + exendin-4 (NT EX4, *P* = 0.0003) and hypothermia + saline (HT SAL, *P* = 0.0171), as well as the combined treatment of hypothermia + exendin-4 (HT EX4, *P* < 0.0001) groups (Fig. 8B). Additionally, combined hypothermia + exendin-4 showed significantly less macroscopic tissue loss when compared to hypothermia treatment + saline (HT SAL, *P* = 0.0532). Exendin-4 single high dose either alone or in combination with hypothermia did not affect weight gain (Fig. 8C). To more accurately assess tissue loss, volume infarction was measured. Normothermia mice demonstrated substantial total volume tissue loss (11.6 ± 1.2 mm^3^). Exendin-4 single high dose (3.9 ± 1.4 mm^3^, *P* = 0.0003), hypothermia alone (5.2 ± 1.2 mm^3^, *P* = 0.0115) and combined hypothermia and exendin-4 (1.1 ± 0.6 mm^3^, *P* < 0.0001) treatments substantially reduced the infarction volume (Fig. 8D). Analysis of volume loss across different brain levels showed that in level 1, NT EX4 and HT EX4 animals were significantly protected (*P* = 0.0067 and *P* < 0.0001). All three treatments resulted in significant protection when assessing levels 2–5: level 2 NT EX4 (*P* = 0.0019), HT SAL (*P* = 0.0466), HT EX4 (*P* < 0.0001); level 3 NT EX4 (*P* = 0.0112), HT SAL (*P* = 0.0287), HT EX4 (*P* < 0.0001); level 4 NT EX4 (*P* = 0.0016), HT SAL (*P* = 0.0527), HT EX4 (*P* < 0.0001); level 5 NT EX4 (*P* = 0.0006), HT SAL (*P* = 0.0206), HT EX4 (*P* < 0.0001) (Fig. 8E). Additionally, HT EX4 animals had substantially less volume loss than HT SAL when assessing levels 1 (*P* = 0.0093) and 2 (*P* = 0.0541).

## Discussion

There is an enormous unmet clinical need for effective interventions against neonatal HIE. We have conducted a preclinical study of the therapeutic efficacy and safety of exendin-4 in a perinatal mouse model of HIE. Here we establish for the first time that exendin-4 treatment after neonatal HIE is highly neuroprotective. Furthermore, we demonstrate that exendin-4 can be used in synergy with hypothermia, the current clinical standard of care for HIE, to enhance its therapeutic efficacy.

In our late preterm results, intraperitoneal administration of 0.5 µg/g exendin-4 as a four-dose 12 h interval regimen started immediately after hypoxia-ischaemia is significantly neuroprotective. Delaying the start of this exendin-4 treatment regimen by 2 h also significantly protects the immature brain with no significant difference in efficacy when compared to immediate administration post-hypoxia-ischaemia. The significant therapeutic efficacy was also measured in other readouts of neuropathology including a reduction of TUNEL+ cell death and microglia/macrophage, astrocytes and endothelial cells activation markers remained low in contrast to untreated hypoxia-ischaemia groups. Our 2 h delayed exendin-4 regimen results are of particular clinical relevance and may signify a potentially extended window of opportunity in which therapeutic exendin-4 could be administered. In the adult murine stroke model using transient middle cerebral artery occlusion, Teramoto *et al.* (2011) were able to retain reduced infarct volume only up to 1 h delayed administration of high dose exendin-4.

This study shows that exendin-4 alone or in combination with therapeutic hypothermia significantly protects the neonatal brain against term HIE. Hypothermia is the only standard treatment of care for term HIE in developed countries and is not used in preterm infants or in developing countries. The latter has a high association with infection, where hypothermia may have limited or even a detrimental effect (Robertson *et al.*, 2008; Osredkar *et al.*, 2014). Furthermore, hypothermia is only partially protective, and alternative or adjunct therapies are needed. Because of the variability in the efficacy of hypothermia in the neonatal rodent model of HIE, we administered a single high dose of exendin-4 in combination with hypothermia to allow clear dissection of a potential synergistic adjunct role between both treatments. Exendin-4 administration provided neuroprotection against tissue loss in the P10 term mouse comparable to the P7 single-dose results, demonstrating that the same dose is applicable for both developmental ages. Hypothermia alone also proved significantly neuroprotective; however, the addition of a single dose of exendin-4 treatment enhanced hypothermia protection both in the macroscopic injury score and regional infarct volume assessments.

Exendin-4 is an FDA and EMA approved drug used clinically for the treatment of type 2 diabetes mellitus (Furman, 2012). Epidemiological studies have established comorbidity links between type 2 diabetes mellitus and several adult onset neurodegenerative and cerebrovascular disorders, where patients with type 2 diabetes mellitus have increased risk of Alzheimer’s disease (Peila *et al.*, 2002), Parkinson’s disease (Santiago and Potashkin, 2013) and stroke (Putaala *et al.*, 2011). This suggests shared disease mechanisms, and inflammation, aberrant insulin and insulin-like growth factor 1 (IGF1) signalling and mitochondria dysfunction have shown to contribute to pathogenesis of these conditions (Kruyt *et al.*, 2010; Donath and Shoelson, 2011; Clark *et al.*, 2012; Aviles-Olmos *et al.*, 2013*b*). Most neonatal studies rely on extensive research of adult experimental models, and despite biological differences, inflammation (Hagberg *et al.*, 2015) and mitochondria dysfunction (Hagberg *et al.*, 2014) are also major hallmarks of HIE. There are other reasonable links between hypoxia-ischaemia and the aforementioned adult disorders. Preclinical rat studies have shown neonatal hypoxia-ischaemia as a precursor to diabetes, metabolic syndrome and stroke (Mcpherson *et al.*, 2009), and IGF1 treatment offers neuroprotection after hypoxia-ischaemia (Brywe *et al.*, 2005). Therefore, exendin-4-mediated prevention of mitochondria damage and stimulation of mitochondria biogenesis (Fan *et al.*, 2010), anti-inflammatory properties and sustained neuroprotection beyond the period of intervention (Athauda *et al.*, 2017) make it an attractive therapeutic approach in the treatment of HIE. In fact, exendin-4 has already been used in neonatal animals to prevent adolescent and adult disorders without adverse effects. Transient neonatal preconditioning with exendin-4 in lamb (Gatford *et al.*, 2013) and rat (Stoffers *et al.*, 2003) intrauterine growth restriction models reduced both visceral fat accumulation, a risk factor for type 2 diabetes mellitus, and oxidative stress (Raab *et al.*, 2009). Neonatal exendin-4 protected against juvenile and adult rat myocardial ischaemic injury and preconditioned mitochondria (Brown *et al.*, 2010). Its administration in neonatal wild-type C57BL/6 mice prevented increased adult body weight and fat mass, and increased energy levels via GLP1R activation (Rozo *et al.*, 2017).

The optimal exendin-4 dose concentration used in this study (0.5 µg/g) is significantly higher than in the treatment of type 2 diabetes mellitus (0.1 µg/kg) and in the ongoing Alzheimer’s and Parkinson’s disease trials. The doses used in this study are based on the findings by Teramoto *et al.* (2011), which required the same dose concentration or higher (10, 50 µg/100µl per mouse) to exert protection against transient middle cerebral artery occlusion (Teramoto *et al.*, 2011). We observed that reducing the dose concentration by 10-fold to 0.05 µg/g resulted in abolition of the exendin-4 neuroprotective effect in the HIE model. This high dose requirement may reflect the acute and rapid nature of the injury. Hypoglycaemia is a serious risk for infants suffering from HIE, and blood glucose is continuously monitored in neonatal intensive care units (Tam *et al.*, 2012). Therefore, we monitored blood glucose at different time points after high dose exendin-4 injection, and the known glucose lowering effects of exendin-4 was not observed when compared to saline-treated/basal level controls. This differs from many studies where exendin-4 was associated with substantial reduction in blood glucose levels. However, many of these preclinical exendin-4 studies use diabetic models, which have altered baseline glucose (Young *et al.*, 1999). Clinically, exendin-4 is given to patients with type 2 diabetes mellitus as an adjunct to metformin, sulfonylurea or basal insulin (Inzucchi *et al.*, 2015), and it is the exendin-4 addition to metformin and/or sulfonylurea that enables type 2 diabetes mellitus patients to achieve glycaemic control via improvement of β-cell function (Kendall *et al.*, 2005). These results are compatible with exendin-4’s positive effect on glucose homeostasis. Moreover, exendin-4 administration in healthy individuals reduces fasting glucose levels without reaching hypoglycaemic levels (Edwards *et al.*, 2001). Our assessment of any adverse effects to this transient systemically administered high dose exendin-4 regimen did not reveal any toxicity through blood, macrophage-inflammatory or histological analyses. Our results indicate that the high dose regimen used in this study is safe and well-tolerated in mice. These findings are supported by a pharmacology and toxicity review of exenatide (Byetta®) reporting that doses of 450 times the clinical dose in normal glycaemic monkeys produces no hypoglycaemia, neurological signs or pathology (https://www.accessdata.fda.gov/drugsatfda_docs/nda/2009/021919s000pharmr.pdf). Chronic toxicity studies in monkeys administered very high doses of up to 1000 µg/kg over a 28-day period resulted in no mortality. Any risks are further mitigated as we have advocated high doses administered once or every 12 h over a limited 48 h period (for optimal therapy) when the pathological cascade associated with HIE is most significant. The lack of commercially available kits to measure exendin-4 makes comparison of plasma levels in this study with those in humans difficult. However, the same review shows that a 200 µg/kg subcutaneous administration in mice leads to a C_max_ value of 318 507 pg/ml compared to a human that had received 10 µg/subject and a C_max_ of 251 pg/ml.

In our study, high dose exendin-4 in post-hypoxic-ischaemic injury or naïve mice resulted in an initial weight drop, with a return to baseline level (0 h time-point equivalent) 48 h after start. However, a 10-fold lower dose of exendin-4 (0.05 µg/g) given four times had no effect on body weight when compared to controls, suggesting that this dose might be too low for exendin-4 to sufficiently activate GLP1R in the brain of neonates. Interestingly, P10 mice treated with one high dose exendin-4 used in conjunction with hypothermia showed no weight change in comparison to controls. This could signify that hypothermia may have a modulating effect on exendin-4-mediated activation of GLP1R, although not enough to diminish its neuroprotective effects in the HIE model.

Exendin-4 was administered intraperitoneally and several studies have shown that it can readily cross the blood–brain barrier (Kastin and Akerstrom, 2003; Zanotto *et al.*, 2017) and interact directly with GLP1R in neural cells. Our measurements of cAMP (a second messenger of GLP1R) in the brain at regular time intervals following a single intraperitoneal administration of exendin-4 to naïve P7 mice showed significantly elevated levels at the earliest 2 h time point. This suggests that exendin-4 rapidly crosses the blood–brain barrier and initiates a pharmacological response. GLP1R is expressed in the human (Wei and Mojsov, 1995), rat (Göke *et al.*, 1995) and mouse (Hamilton and Hölscher, 2009) brain. Similarly, we have shown ubiquitous expression of GLP1R throughout the naïve mouse brain irrespective of developmental age (P7, P10 and 10 weeks) and confirm for the first time in the human preterm neonatal brain. In the neonatal period (P7 and P10) GLP1R seemed to predominantly co-localize with neuronal cells, whereas GLP1R in adult mice also showed co-localization with astroglia. We believe that this is the first study to confirm GLP1R expression in the neonatal mouse brain, and its main co-localization with neurons at this developmental stage.

The precise mechanisms of action of exendin-4 neuroprotection are still not fully understood. Several studies have demonstrated a multitude of neuroprotective actions, with exendin-4-mediated PI3K surge in different areas of the CNS, leading to increase in phosphorylation of AKT via PI3K signalling pathway. This mechanism of action is thought to increase the anti- versus pro-apoptotic bcl-2 family protein balance (Brywe *et al.*, 2005; Athauda and Foltynie, 2016), attenuate neuroinflammation and stabilize the blood–brain barrier post-transient middle cerebral artery occlusion in non- (Chen *et al.*, 2016) and diabetic mice (Li *et al.*, 2016). Intracellular cAMP levels are also raised in exendin-4-treated post-transient middle cerebral artery occlusion as a result of increased GLP1R expression (Teramoto *et al.*, 2011; Kim *et al.*, 2017). Exendin-4 administration also increases islet-brain 1, partially inhibits JNK and attenuates downstream COX-2 and prostaglandin E_2_ after transient middle cerebral artery occlusion (Kim *et al.*, 2017). Exendin-4 treatment has resulted in mitochondria biogenesis (Fan *et al.*, 2010; Kang *et al.*, 2015), protection from reactive oxygen species (Teramoto *et al.*, 2011; Li *et al.*, 2013), inflammatory inhibition (Teramoto *et al.*, 2011), neurogenesis (Bertilsson *et al.*, 2008), neurotrophic effects (Perry *et al.*, 2002) and cell survival (Teramoto *et al.*, 2011; Candeias *et al.*, 2017). These neuroprotective effects are of extreme importance in offsetting the mechanisms of HIE injury.

The current study was designed to assess exendin-4 drug preclinical safety and efficacy as a standalone therapy, its capacity to augment therapeutic hypothermia neuroprotection, and not specifically the exendin-4 mechanisms of action. However, aspects of our study suggest that exendin-4 may act to maintain neuronal viability and modulate neuroinflammation. Microglia, astroglia and endothelial cells are a source of inflammatory response and reactive oxygen species after HIE (Dietrich, 2002; Hagberg *et al.*, 2015; Rocha-Ferreira *et al.*, 2016). In our study, four high dose treatments of exendin-4 started either immediately or 2 h post-hypoxia-ischaemia significantly reduced proliferation of microglia and its morphological change into phagocytic amoeboid microglia. GFAP expression in astrocytes also remained low and there was reduced ICAM1 immunoreactivity. ICAM1 upregulation is associated with diapedesis, transendothelial migration and further recruitment of peripheral inflammatory cells (Dietrich, 2002). In neonatal hypoxia-ischaemia, ICAM1 is also associated with alterations of the brain microvasculature and breakdown of the blood–brain barrier (Lai *et al.*, 2017). Our ICAM1 results showed increased ICAM1immunoreactivity, particularly in the areas surrounding the tissue infarction observed in the hypoxia-ischaemia group, an effect not observed in the exendin-4-treated groups. This suggests that exendin-4 treatment prevents breakdown of the blood–brain barrier. This is in agreement with a study by Zanotto *et al.* (2017) where exendin-4 treatment reversed blood–brain barrier permeability and reduction of blood–brain barrier-specific proteins in diabetes mellitus rats. The suggested beneficial effect of reduced activation of these cells occurs in parallel with significantly reduced tissue infarction assessed through Nissl staining and cell death (TUNEL assay) in exendin-4-treated mice. The neuroprotective effect of exendin-4 alone seemed moderately long-lasting, as seen in the P10 single dose treatment part of the study. Our studies showed predominant neuronal expression of GLP1R. Therefore, it cannot be excluded that the reduced glial cell activation may be secondary to reduced neurodegeneration. Additionally, high dose exendin-4 administration resulted in significantly higher levels of brain cAMP, suggesting involvement of the cAMP signalling pathway, also proposed in other studies (Teramoto *et al.*, 2011; Kim *et al.*, 2017). Interestingly, metformin, another type 2 diabetes mellitus medication with diverse pharmacological activities, also ameliorates brain infarction in neonatal hypoxic-ischaemic rats (Fang *et al.*, 2017).

This preclinical study in a mouse model of acute perinatal HIE is an essential first step to potentially advance the use of exendin-4 for clinical benefit. Using severe hypoxic-ischaemic injury in both the late preterm and term models has shown exendin-4’s efficacy in both ages. Preterm treatment with exendin-4 alone is highly attractive as therapeutic hypothermia is not common practise at this age, and the successful 2-h delayed start of exendin-4 treatment offers a larger therapeutic window of opportunity within which to administer the drug. Exendin-4 term treatment combined with therapeutic hypothermia has provided enhanced protection as seen in the brain macroscopic score and regional volume measurement. Other proof of concept studies of exendin-4 have already been translated into early clinical trials for neurological diseases such as Alzheimer’s disease (NCT01255163) and, in particular, Parkinson’s disease for which initial reports are highly promising (NCT01174810; NCT01971242; Aviles-Olmos *et al.*, 2013*a*; Athauda *et al.*, 2017). This study and these early clinical trials support the continued investigation of exendin-4 for clinical translation as a treatment for HIE.

## Supplementary Material

Supplementary Figures-Tables

## Abbreviations

GLP-1: glucagon-like peptide-1
HIE: hypoxic-ischaemic encephalopathy
TUNEL: terminal deoxynucleotidyl transferase dUTP nick-end labelling

## References

[awy220-B1] Athauda D, Foltynie T The glucagon-like peptide 1 (GLP) receptor as a therapeutic target in Parkinson’s disease: mechanisms of action. Drug Discov Today 2016; 21: 802–18. 26851597 10.1016/j.drudis.2016.01.013

[awy220-B2] Athauda D, Maclagan K, Skene SS, Bajwa-Joseph M, Letchford D, Chowdhury K et al Exenatide once weekly versus placebo in Parkinson’s disease: a randomised, double-blind, placebo-controlled trial. Lancet 2017; 390: 1664–75. 28781108 10.1016/S0140-6736(17)31585-4 PMC5831666

[awy220-B3] Aviles-Olmos I, Dickson J, Kefalopoulou Z, Djamshidian A, Ell P, Soderlund T et al Exenatide and the treatment of patients with Parkinson’s disease. J Clin Invest2013a; 123: 2730–6. 23728174 10.1172/JCI68295 PMC3668846

[awy220-B4] Aviles-Olmos I, Limousin P, Lees A, Foltynie T Parkinson’s disease, insulin resistance and novel agents of neuroprotection. Brain2013b; 136: 374–84. 22344583 10.1093/brain/aws009

[awy220-B5] Bertilsson G, Patrone C, Zachrisson O, Andersson A, Dannaeus K, Heidrich J et al Peptide hormone exendin-4 stimulates subventricular zone neurogenesis in the adult rodent brain and induces recovery in an animal model of Parkinson’s disease. J Neurosci Res 2008; 86: 326–38. 17803225 10.1002/jnr.21483

[awy220-B6] Blumberg RM, Cady EB, Wigglesworth JS, McKenzie JE, Edwards AD Relation between delayed impairment of cerebral energy metabolism and infarction following transient focal hypoxia-ischaemia in the developing brain. Exp Brain Res 1997; 113: 130–7. 9028781 10.1007/BF02454148

[awy220-B7] Brown SB, Libonati JR, Selak MA, Shannon RP, Simmons RA Neonatal Exendin-4 leads to protection from reperfusion injury and reduced rates of oxidative phosphorylation in the adult rat heart. Cardiovasc Drugs Ther 2010; 24: 197–205. 20582459 10.1007/s10557-010-6242-z PMC4492538

[awy220-B8] Brywe KG, Mallard C, Gustavsson M, Hedtjärn M, Leverin AL, Wang X et al IGF-I neuroprotection in the immature brain after hypoxia-ischemia, involvement of Akt and GSK3β? Eur J Neurosci 2005; 21: 1489–502. 15845077 10.1111/j.1460-9568.2005.03982.x

[awy220-B9] Candeias E, Sebastião I, Cardoso S, Carvalho C, Santos MS, Oliveira CR et al Brain GLP-1/IGF-1 signaling and autophagy mediate exendin-4 protection against apoptosis in type 2 diabetic rats. Mol Neurobiol 2017; 55: 4030–50. 28573460 10.1007/s12035-017-0622-3

[awy220-B10] Carlsson Y, Schwendimann L, Vontell R, Rousset CI, Wang X, Lebon S et al Genetic inhibition of caspase-2 reduces hypoxic-ischemic and excitotoxic neonatal brain injury. Ann Neurol 2011; 70: 781–9. 21674587 10.1002/ana.22431

[awy220-B11] Carlsson Y, Wang X, Schwendimann L, Rousset CI, Jacotot E, Gressens P et al Combined effect of hypothermia and caspase-2 gene deficiency on neonatal hypoxic-ischemic brain injury. Pediatr Res 2012; 71: 566–72. 22322383 10.1038/pr.2012.15

[awy220-B12] Chen F, Wang W, Ding H, Yang Q, Dong Q, Cui M The glucagon-like peptide-1 receptor agonist exendin-4 ameliorates warfarin-associated hemorrhagic transformation after cerebral ischemia. J Neuroinflammation 2016; 13: 204. 27566245 10.1186/s12974-016-0661-0 PMC5002167

[awy220-B13] Clark I, Atwood C, Bowen R, Paz-Filho G, Vissel B Tumor necrosis factor-induced cerebral insulin resistance in Alzheimer’s disease links numerous treatment rationales. Pharmacol Rev 2012; 64: 1004–26. 22966039 10.1124/pr.112.005850

[awy220-B14] Deacon CF, Pridal L, Klarskov L, Olesen M, Holst JJ Glucagon-like peptide-1 undergoes differential tissue- specific metabolism in the anesthetized pig. Am J Physiol Metab 1996; 271 (3 Pt 1): E458–64. 10.1152/ajpendo.1996.271.3.E458 8843738

[awy220-B15] Dietrich JB The adhesion molecule ICAM-1 and its regulation in relation with the blood-brain barrier. J Neuroimmunol 2002; 128: 58–68. 12098511 10.1016/s0165-5728(02)00114-5

[awy220-B16] Donath MY, Shoelson SE Type 2 diabetes as an inflammatory disease. Nat Rev Immunol 2011; 11: 98–107. 21233852 10.1038/nri2925

[awy220-B17] Drucker DJ Incretin action in the pancreas: potential promise, possible perils, and pathological pitfalls. Diabetes 2013; 62: 3316–23. 23818527 10.2337/db13-0822 PMC3781450

[awy220-B18] Dupont WD, Plummer WD Power and sample size calculations. A review and computer program. Control Clin Trials 1990; 11: 116–28. 2161310 10.1016/0197-2456(90)90005-m

[awy220-B19] Dupont WD, Plummer WD Power and sample size calculations for studies involving linear regression. Control Clin Trials 1998; 19: 589–601. 9875838 10.1016/s0197-2456(98)00037-3

[awy220-B20] Edwards AD, Brocklehurst P, Gunn AJ, Halliday H, Juszczak E, Levene M et al Neurological outcomes at 18 months of age after moderate hypothermia for perinatal hypoxic ischaemic encephalopathy: synthesis and meta-analysis of trial data. BMJ 2010; 340: 409. 10.1136/bmj.c363 PMC2819259 20144981

[awy220-B21] Edwards CM, Stanley SA, Davis R, Brynes AE, Frost GS, Seal LJ et al Exendin-4 reduces fasting and postprandial glucose and decreases energy intake in healthy volunteers. Am J Physiol Endocrinol Metab 2001; 281: E155–61. 11404233 10.1152/ajpendo.2001.281.1.E155

[awy220-B22] Fan R, Li X, Gu X, Chan JCN, Xu G Exendin-4 protects pancreatic beta cells from human islet amyloid polypeptide-induced cell damage: potential involvement of AKT and mitochondria biogenesis. Diabetes Obes Metab 2010; 12: 815–24. 20649634 10.1111/j.1463-1326.2010.01238.x

[awy220-B23] Fang M, Jiang H, Ye L, Cai C, Hu Y, Pan S et al Metformin treatment after the hypoxia-ischemia attenuates brain injury in newborn rats. Oncotarget 2017; 8: 75308–25. 29088867 10.18632/oncotarget.20779 PMC5650422

[awy220-B24] Furman BL The development of Byetta (exenatide) from the venom of the Gila monster as an anti-diabetic agent. Toxicon 2012; 59: 464–71. 21194543 10.1016/j.toxicon.2010.12.016

[awy220-B25] Gatford KL, Sulaiman SA, Mohammad SN, De Blasio MJ, Harland ML, Simmons RA et al Neonatal exendin-4 reduces growth, fat deposition and glucose tolerance during treatment in the intrauterine growth-restricted lamb. PLoS One 2013; 8: e56553. 23424667 10.1371/journal.pone.0056553 PMC3570470

[awy220-B26] Gilland E, Bona E, Hagberg H Temporal changes of regional glucose use, blood flow, and microtubule-associated protein 2 immunostaining after hypoxia-ischemia in the immature rat brain. J Cereb Blood Flow Metab1998a; 18: 222–8. 9469166 10.1097/00004647-199802000-00014

[awy220-B27] Gilland E, Puka-Sundvall M, Hillered L, Hagberg H Mitochondrial function and energy metabolism after hypoxia-ischemia in the immature rat brain: involvement of NMDA-receptors. J Cereb Blood Flow Metab1998b; 18: 297–304. 9498846 10.1097/00004647-199803000-00008

[awy220-B28] Gluckman PD, Wyatt JS, Azzopardi D, Ballard R, Edwards AD, Ferriero DM et al Selective head cooling with mild systemic hypothermia after neonatal encephalopathy: multicentre randomised trial. Lancet 2005; 365: 663–70. 15721471 10.1016/S0140-6736(05)17946-X

[awy220-B29] Göke R, Larsen PJ, Mikkelsen JD, Sheikh SP Distribution of GLP‐1 binding sites in the rat brain: evidence that exendin‐4 is a ligand of brain GLP‐1 binding sites. Eur J Neurosci 1995; 7: 2294–300. 8563978 10.1111/j.1460-9568.1995.tb00650.x

[awy220-B30] Grant P, Lipscomb D, Quin J Psychological and quality of life changes in patients using GLP-1 analogues. J Diabetes Complications 2011; 25: 244–6. 21601480 10.1016/j.jdiacomp.2011.03.002

[awy220-B31] Hagberg H, Mallard C, Ferriero DM, Vannucci SJ, Levison SW, Vexler ZS et al The role of inflammation in perinatal brain injury. Nat Rev Neurol 2015; 11: 192–208. 25686754 10.1038/nrneurol.2015.13 PMC4664161

[awy220-B32] Hagberg H, Mallard C, Rousset CI, Thornton C Mitochondria: hub of injury responses in the developing brain. Lancet Neurol 2014; 13: 217–32. 24457191 10.1016/S1474-4422(13)70261-8

[awy220-B33] Hamilton A, Hölscher C Receptors for the incretin glucagon-like peptide-1 are expressed on neurons in the central nervous system. Neuroreport 2009; 20: 1161–6. 19617854 10.1097/WNR.0b013e32832fbf14

[awy220-B34] He L, Law PTY, Wong CK, Chan JCN, Chan PKS Exendin-4 exhibits enhanced anti-tumor effects in diabetic mice. Sci Rep 2017; 7: 1791. 28496193 10.1038/s41598-017-01952-5 PMC5431757

[awy220-B35] Inamasu J, Suga S, Sato S, Horiguchi T, Akaji K, Mayanagi K et al Post-ischemic hypothermia delayed neutrophil accumulation and microglial activation following transient focal ischemia in rats. J Neuroimmunol 2000; 109: 66–74. 10996208 10.1016/s0165-5728(00)00211-3

[awy220-B36] Inzucchi SE, Bergenstal RM, Buse JB, Diamant M, Ferrannini E, Nauck M et al Management of hyperglycemia in type 2 diabetes, 2015: a patient-centered approach: update to a position statement of the american diabetes association and the european association for the study of diabetes. Diabetes Care 2015; 38: 140–9. 25538310 10.2337/dc14-2441

[awy220-B37] Johnston MV, Fatemi A, Wilson MA, Northington F Treatment advances in neonatal neuroprotection and neurointensive care. Lancet Neurol 2011; 10: 372–82. 21435600 10.1016/S1474-4422(11)70016-3 PMC3757153

[awy220-B38] Kang MY, Oh TJ, Cho YM Glucagon-like peptide-1 increases mitochondrial biogenesis and function in INS-1 rat insulinoma cells. Endocrinol Metab 2015; 30: 216–20. 10.3803/EnM.2015.30.2.216 PMC4508267 26194081

[awy220-B39] Kastin AJ, Akerstrom V Entry of exendin-4 into brain is rapid but may be limited at high doses. Int J Obes 2003; 27: 313–18. 10.1038/sj.ijo.0802206 12629557

[awy220-B40] Kendall DM, Riddle MC, Rosenstock J, Zhuang D, Kim DD, Fineman MS et al Effects of exenatide (exendin-4) on glycemic control over 30 weeks in patients with type 2 diabetes treated with metformin and a sulfonylurea. Diabetes Care 2005; 28: 1083–91. 15855571 10.2337/diacare.28.5.1083

[awy220-B41] Kilkenny C, Browne W, Cuthill IC, Emerson M, Altman DG Editorial: animal research: reporting *in vivo* experiments-The ARRIVE Guidelines. J Cereb Blood Flow Metab 2011; 31: 991–3. 21206507 10.1038/jcbfm.2010.220 PMC3070981

[awy220-B42] Kim S, Jeong J, Jung H-S, Kim B, Kim Y-E, Lim D-S et al Anti-inflammatory effect of glucagon like peptide-1 receptor agonist, Exendin-4, through modulation of IB1/JIP1 expression and JNK signaling in stroke. Exp Neurobiol 2017; 26: 227–39. 28912645 10.5607/en.2017.26.4.227 PMC5597553

[awy220-B43] Kruyt ND, Biessels GJ, Devries JH, Roos YB Hyperglycemia in acute ischemic stroke: pathophysiology and clinical management. Nat Rev Neurol 2010; 6: 145–55. 20157308 10.1038/nrneurol.2009.231

[awy220-B44] Lai JCY, Rocha-Ferreira E, Ek CJ, Wang X, Hagberg H, Mallard C Immune responses in perinatal brain injury. Brain Behav Immun 2017; 63: 210–23. 27865947 10.1016/j.bbi.2016.10.022

[awy220-B45] Lee AC, Kozuki N, Blencowe H, Vos T, Bahalim A, Darmstadt GL et al Intrapartum-related neonatal encephalopathy incidence and impairment at regional and global levels for 2010 with trends from 1990. Pediatr Res 2013; 74 (Suppl 1): 50–72. 24366463 10.1038/pr.2013.206 PMC3873711

[awy220-B46] Li P-C, Liu L-F, Jou M-J, Wang H-K The GLP-1 receptor agonists exendin-4 and liraglutide alleviate oxidative stress and cognitive and micturition deficits induced by middle cerebral artery occlusion in diabetic mice. BMC Neurosci 2016; 17: 37. 27296974 10.1186/s12868-016-0272-9 PMC4907076

[awy220-B47] Li Z, Zhou Z, Huang G, Hu F, Xiang Y, He L Exendin-4 protects mitochondria from reactive oxygen species induced apoptosis in pancreatic beta cells. PLoS One 2013; 8: e76172. 24204601 10.1371/journal.pone.0076172 PMC3811987

[awy220-B48] Mcpherson RJ, Mascher-Denen M, Juul SE Postnatal stress produces hyperglycemia in adult rats exposed to hypoxia-ischemia. Pediatr Res 2009; 66: 278–82. 19531978 10.1203/PDR.0b013e3181b1bd1b

[awy220-B49] Nielsen LL, Young AA, Parkes DG Pharmacology of exenatide (synthetic exendin - 4): a potential therapeutic for improved glycemic control of type 2 diabetes. Elsevier Regul Pept 2004; 117: 77–88. 10.1016/j.regpep.2003.10.028 14700743

[awy220-B50] Nijboer CH, Heijnen CJ, van der Kooij MA, Zijlstra J, van Velthoven CT, Culmsee C et al Targeting the p53 pathway to protect the neonatal ischemic brain. Ann Neurol 2011; 70: 255–64. 21674585 10.1002/ana.22413

[awy220-B51] Osredkar D, Thoresen M, Maes E, Flatebø T, Elstad M, Sabir H Hypothermia is not neuroprotective after infection-sensitized neonatal hypoxic-ischemic brain injury. Resuscitation 2014; 85: 567–72. 24361672 10.1016/j.resuscitation.2013.12.006

[awy220-B52] Peila R, Rodriguez BL, Launer LJ Type 2 diabetes, APOE gene, and the risk for dementia and related pathologies: the Honolulu-Asia Aging Study. Diabetes 2002; 51: 1256–62. 11916953 10.2337/diabetes.51.4.1256

[awy220-B53] Perry T, Lahiri DK, Chen D, Zhou J, Shaw KT, Egan JM et al A novel neurotrophic property of glucagon-like peptide 1: a promoter of nerve growth factor-mediated differentiation in PC12 cells. J Pharmacol Exp Ther 2002; 300: 958–66. 11861804 10.1124/jpet.300.3.958

[awy220-B54] Putaala J, Liebkind R, Gordin D, Thorn LM, Haapaniemi E, Forsblom C et al Diabetes mellitus and ischemic stroke in the young: clinical features and long-term prognosis. Neurology 2011; 76: 1831–7. 21606455 10.1212/WNL.0b013e31821cccc2

[awy220-B55] Raab EL, Vuguin PM, Stoffers DA, Simmons RA Neonatal exendin-4 treatment reduces oxidative stress and prevents hepatic insulin resistance in intrauterine growth-retarded rats. AJP Regul Integr Comp Physiol 2009; 297: R1785–94. 10.1152/ajpregu.00519.2009 PMC2803622 19846744

[awy220-B56] Robertson NJ, Nakakeeto M, Hagmann C, Cowan FM, Acolet D, Iwata O et al Therapeutic hypothermia for birth asphyxia in low-resource settings: a pilot randomised controlled trial. Lancet 2008; 372: 801–3. 18774411 10.1016/S0140-6736(08)61329-X

[awy220-B57] Rocha-Ferreira E, Phillips E, Francesch-Domenech E, Thei L, Peebles DM, Raivich G et al The role of different strain backgrounds in bacterial endotoxin-mediated sensitization to neonatal hypoxic–ischemic brain damage. Neuroscience 2015; 311: 292–307. 26515746 10.1016/j.neuroscience.2015.10.035 PMC4675086

[awy220-B58] Rocha-Ferreira E, Rudge B, Hughes MP, Rahim AA, Hristova M, Robertson NJ Immediate remote ischemic postconditioning reduces brain nitrotyrosine formation in a piglet asphyxia model. Oxid Med Cell Longev 2016; 2016: 5763743. 27379176 10.1155/2016/5763743 PMC4917706

[awy220-B59] Rosomoff H, Holaday D Cerebral blood flow and cerebral oxygen consumption during hypothermia. Am J Physiol 1954; 179: 85–8. 13207391 10.1152/ajplegacy.1954.179.1.85

[awy220-B60] Rozo AV, Babu DA, Suen PM, Groff DN, Seeley RJ, Simmons RA et al Neonatal GLP1R activation limits adult adiposity by durably altering hypothalamic architecture. Mol Metab 2017; 6: 748–59. 28702330 10.1016/j.molmet.2017.05.006 PMC5485307

[awy220-B61] Santiago JA, Potashkin JA Shared dysregulated pathways lead to Parkinson’s disease and diabetes. Trends Mol Med 2013; 19: 176–86. 23375873 10.1016/j.molmed.2013.01.002

[awy220-B62] Semple BD, Blomgren K, Gimlin K, Ferriero DM, Noble-Haeusslein LJ Brain development in rodents and humans: identifying benchmarks of maturation and vulnerability to injury across species. Prog Neurobiol 2013; 106–107: 1–16. 10.1016/j.pneurobio.2013.04.001 PMC3737272 23583307

[awy220-B63] Shankaran S Hypoxic-ischemic encephalopathy and novel strategies for neuroprotection. Clin Perinatol 2012; 39: 919–29. 23164187 10.1016/j.clp.2012.09.008

[awy220-B64] Stoffers DA, Desai BM, DeLeon DD, Simmons RA Neonatal exendin-4 prevents the development of diabetes in the intrauterine growth retarded rat. Diabetes 2003; 52: 734–40. 12606515 10.2337/diabetes.52.3.734

[awy220-B65] Stridh L, Mottahedin A, Johansson ME, Valdez RC, Northington F, Wang X et al Toll-like receptor-3 activation increases the vulnerability of the neonatal brain to hypoxia-ischemia. J Neurosci 2013; 33: 12041–51. 23864690 10.1523/JNEUROSCI.0673-13.2013 PMC3713735

[awy220-B66] Tam EW, Haeusslein LA, Bonifacio SL, Glass HC, Rogers EE, Jeremy RJ et al Hypoglycemia is associated with increased risk for brain injury and adverse neurodevelopmental outcome in neonates at risk for encephalopathy. J Pediatr 2012; 161: 88–93. 22306045 10.1016/j.jpeds.2011.12.047 PMC3346850

[awy220-B67] Teramoto S, Miyamoto N, Yatomi K, Tanaka Y, Oishi H, Arai H et al Exendin-4, a glucagon-like peptide-1 receptor agonist, provides neuroprotection in mice transient focal cerebral ischemia. J Cereb Blood Flow Metab 2011; 31: 1696–705. 21487412 10.1038/jcbfm.2011.51 PMC3170947

[awy220-B68] Tews D, Lehr S, Hartwig S, Osmers A, Paslack W, Eckel J Anti-apoptotic action of exendin-4 in INS-1 beta cells: comparative protein pattern analysis of isolated mitochondria. Horm Metab Res 2009; 41: 294–301. 19085810 10.1055/s-0028-1105911

[awy220-B69] Thoresen M, Bågenholm R, Løberg EM, Apriccna F The stress of being restrained reduces brain damage after a hypoxic-ischaemic insult in the 7-day-old rat. Neuroreport1996a; 7: 481–4. 8730810 10.1097/00001756-199601310-00025

[awy220-B70] Thoresen M, Bagenholm R, Loberg EM, Apricena F, Kjellmer I Posthypoxic cooling of neonatal rats provides protection against brain injury. Arch Dis Child Fetal Neonatal Ed1996b; 74: F3–9. 8653432 10.1136/fn.74.1.f3 PMC2528334

[awy220-B71] Thoresen M, Penrice J, Lorek A, Cady EB, Wylezinska M, Kirkbride V et al Mild hypothermia after severe transient hypoxia-ischemia ameliorates delayed cerebral energy failure in the newborn piglet. Pediatr Res 1995; 37: 667–70. 7603788 10.1203/00006450-199505000-00019

[awy220-B200] Thoresen M, Satas S, Puka-Sundvall M, Whitelaw A, Hallström A, Løberg EM et al Post-hypoxic hypothermia reduces cerebrocortical release of NO and excitotoxins. Neuroreport 1997; 8: 3359–62. 9351672 10.1097/00001756-199710200-00033

[awy220-B72] Wang X, Han W, Du X, Zhu C, Carlsson Y, Mallard C et al Neuroprotective effect of Bax-inhibiting peptide on neonatal brain injury. Stroke 2010; 41: 2050–5. 20671246 10.1161/STROKEAHA.110.589051

[awy220-B73] Wei Y, Mojsov S Tissue-specific expression of the human receptor for glucagon- like peptide-I: brain, heart and pancreatic forms have the same deduced amino acid sequences. FEBS Lett 1995; 358: 219–24. 7843404 10.1016/0014-5793(94)01430-9

[awy220-B74] Yanay O, Bailey AL, Kernan K, Zimmerman JJ, Osborne WR Effects of exendin-4, a glucagon like peptide-1 receptor agonist, on neutrophil count and inflammatory cytokines in a rat model of endotoxemia. J Inflamm Res 2015; 8: 129–35. 26244029 10.2147/JIR.S84993 PMC4521677

[awy220-B75] Young AA, Gedulin BR, Bhavsar S, Bodkin N, Jodka C, Hansen B et al Glucose-lowering and insulin-sensitizing actions of exendin-4: studies in obese diabetic (ob/ob, db/db) mice, diabetic fatty Zucker rats, and diabetic rhesus monkeys (Macaca mulatta). Diabetes 1999; 48: 1026–34. 10331407 10.2337/diabetes.48.5.1026

[awy220-B76] Zanotto C, Simão F, Gasparin MS, Biasibetti R, Tortorelli LS, Nardin P et al Exendin-4 reverses biochemical and functional alterations in the blood–brain and blood–CSF barriers in diabetic rats. Mol Neurobiol 2017; 54: 2154–66. 26927659 10.1007/s12035-016-9798-1

