# Supplementary material for: Neuroprotective exendin-4 enhances hypothermia therapy in a model of hypoxic-ischaemic encephalopathy
Source: Brain. 2018 Aug 24;141(10):2925–42. doi: 10.1093/brain/awy220 (PMC6158761; doi:10.1093/brain/awy220)
Supplement: Supplementary Figures-Tables [file awy220_supplementary_figures-tables.pdf]

Supplementary figures

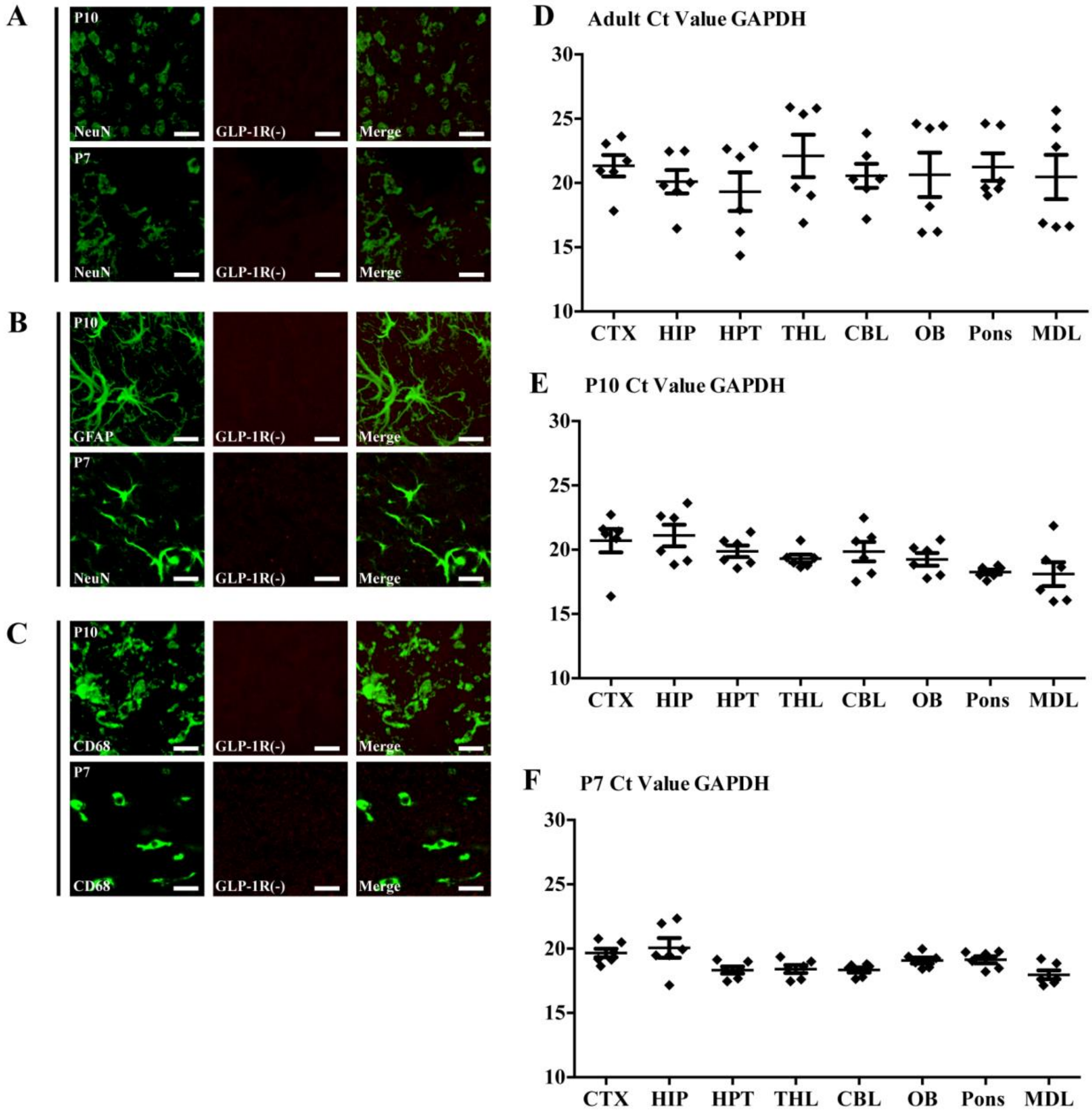

Supplementary figure 1 Temperature chart measurement during 5h of normothermia/ hypothermia immediately after P10 hypoxia-ischaemia insult. (A) One probe in each normothermia and hypothermia sentinel per experimental run measured core temperature using a rectal probe in a total of 8 repeat experiments. Target temperature of 36 degrees for normothermia (red) and 33 degrees for hypothermia (blue). **(B)** One probe in each normothermia (red) and hypothermia (blue) chambers and in the room (black) measured environmental temperature. Data presented as individual animals or as mean ± SEM. Kruskal-Wallis Dunn's test. Scale bar = 2mm.

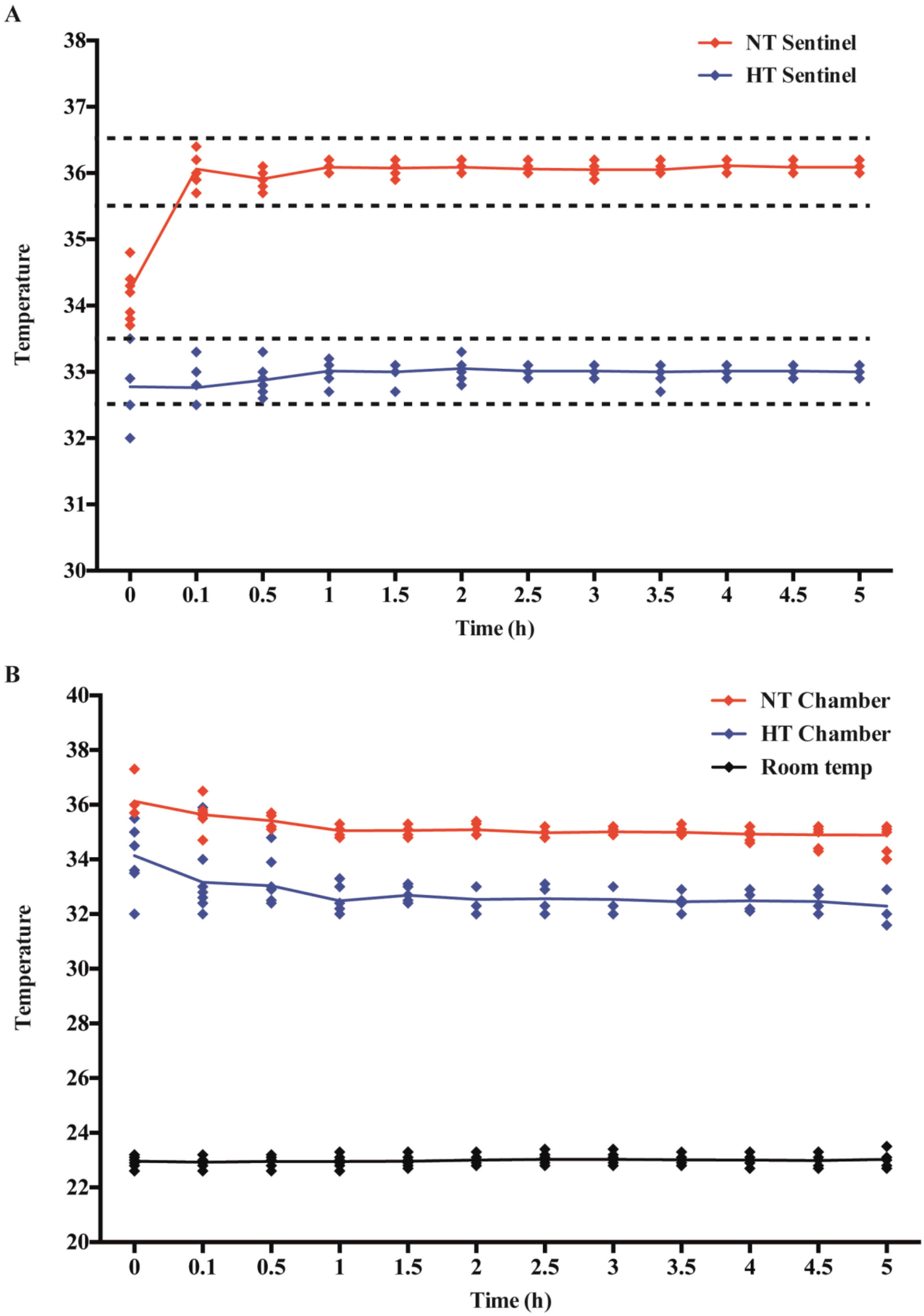

Supplementary figure 2 GLP1R control immunofluorescence and GAPDH qPCR Ct values. (A) Confocal micrographs stained for neurons, **(B)** astrocytes and **(C)** microglia

showing negative staining for GLP1R antibody during the neonatal period (P10 and P7) (Scale bar = 20µm). A median filter was applied to the images to reduce noise. Control GAPDH Ct values of mRNA used for normalisation of GLP1R pPCR data at **(D)** 10 weeks, **(E)** P10 and **(F)** P7. Abbreviations: CTX- cortex, HIP – hippocampus, HPT – hypothalamus, THL – thalamus, CBL – cerebellum, OB – olfactory bulbs, MDL – medulla.

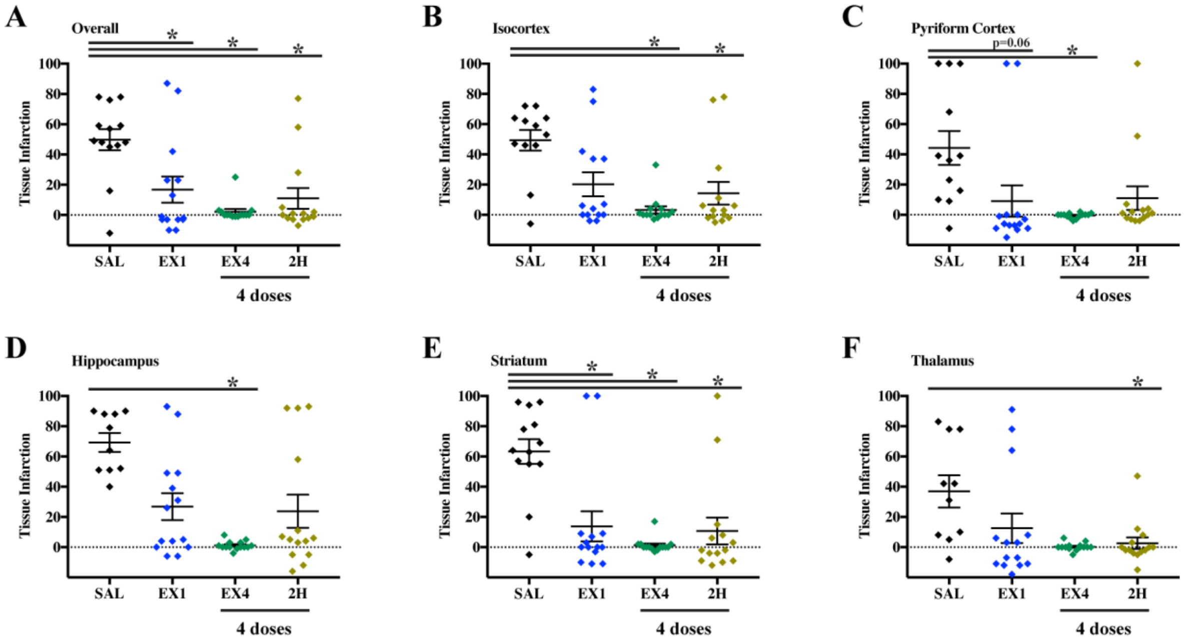

Supplementary figure 3 Regional neuroprotective effect of high-dose exendin-4. (A) Nissl staining assessment of overall tissue infarction (Nissl staining), as well as regional: **(B)** isocortex, **(C)** pyriform cortex, **(D)** hippocampus, **(E)** striatum and **(F)** thalamus brain regions, across the different treatments – saline controls, 1 high-dose exendin-4, four high-doses exendin-4 and four low-doses exendin-4, all started immediately after hypoxia-ischaemia, and four high-doses exeIdin-4 initated 2h after hypoxia-ischaemia. Data presented as individual animals ± SEM and analysed using Kruskal-Wallis Dunn's test. * $P< 0.05$, ** $P< 0.01$, *** $P < 0.001$ and **** $P < 0.0001$.

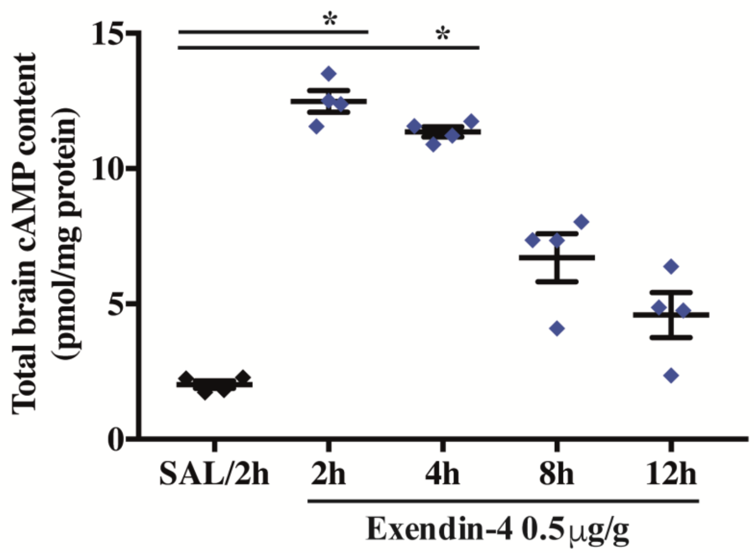

Supplementary figure 4 cAMP. cAMP brain levels in saline and 1 high-dose exendin-4 treated mice at different time-points. Data presented as individual animals ± SEM and analysed using Kruskal-Wallis Dunn's test. * $P< 0.05$, ** $P< 0.01$, *** $P < 0.001$ and **** $P < 0.0001$.

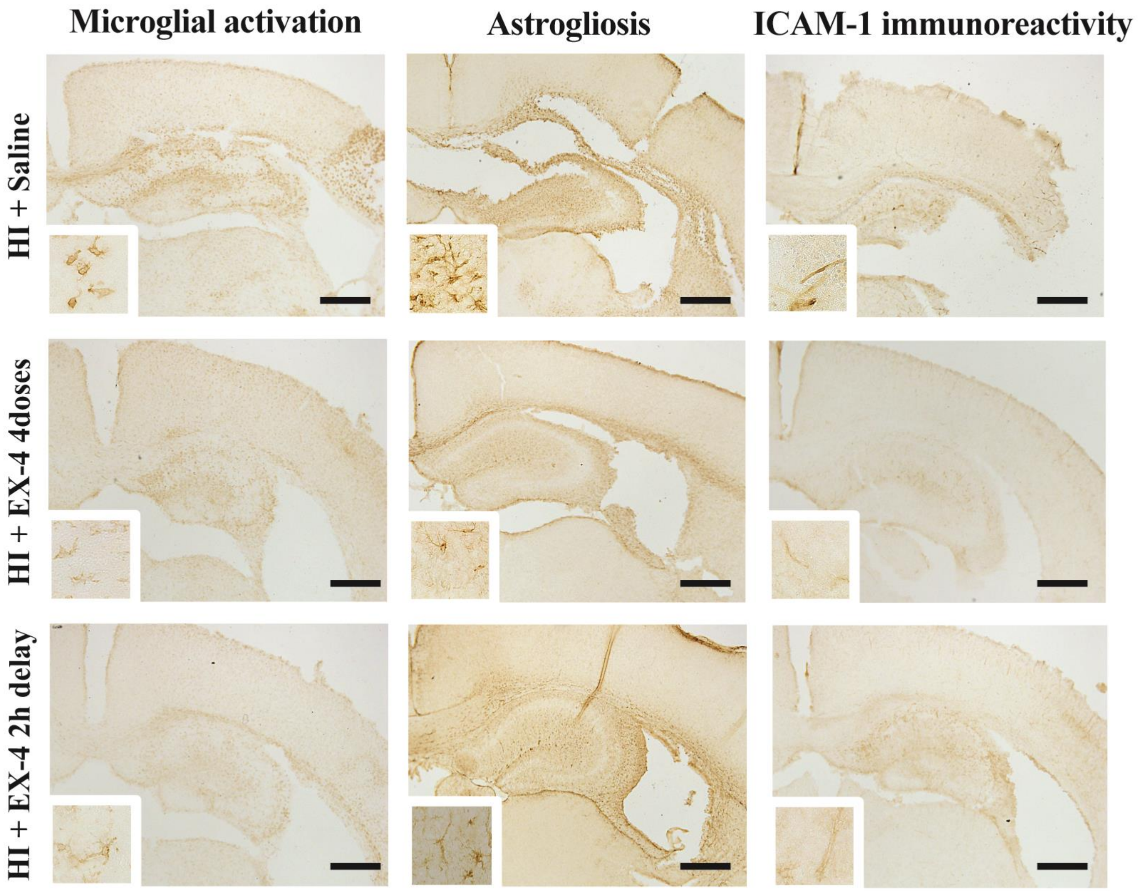

Supplementary figure 5 Glia micrographs. Representative brain micrographs of saline controls, and four high-doses of exendin-4 treatments started either immediately or 2h after hypoxia-ischaemia. Brain sections were stained with alphaM (microglia), GFAP (astrocytes) and ICAM1 (endothelial cells) Scale bar = 400µm.

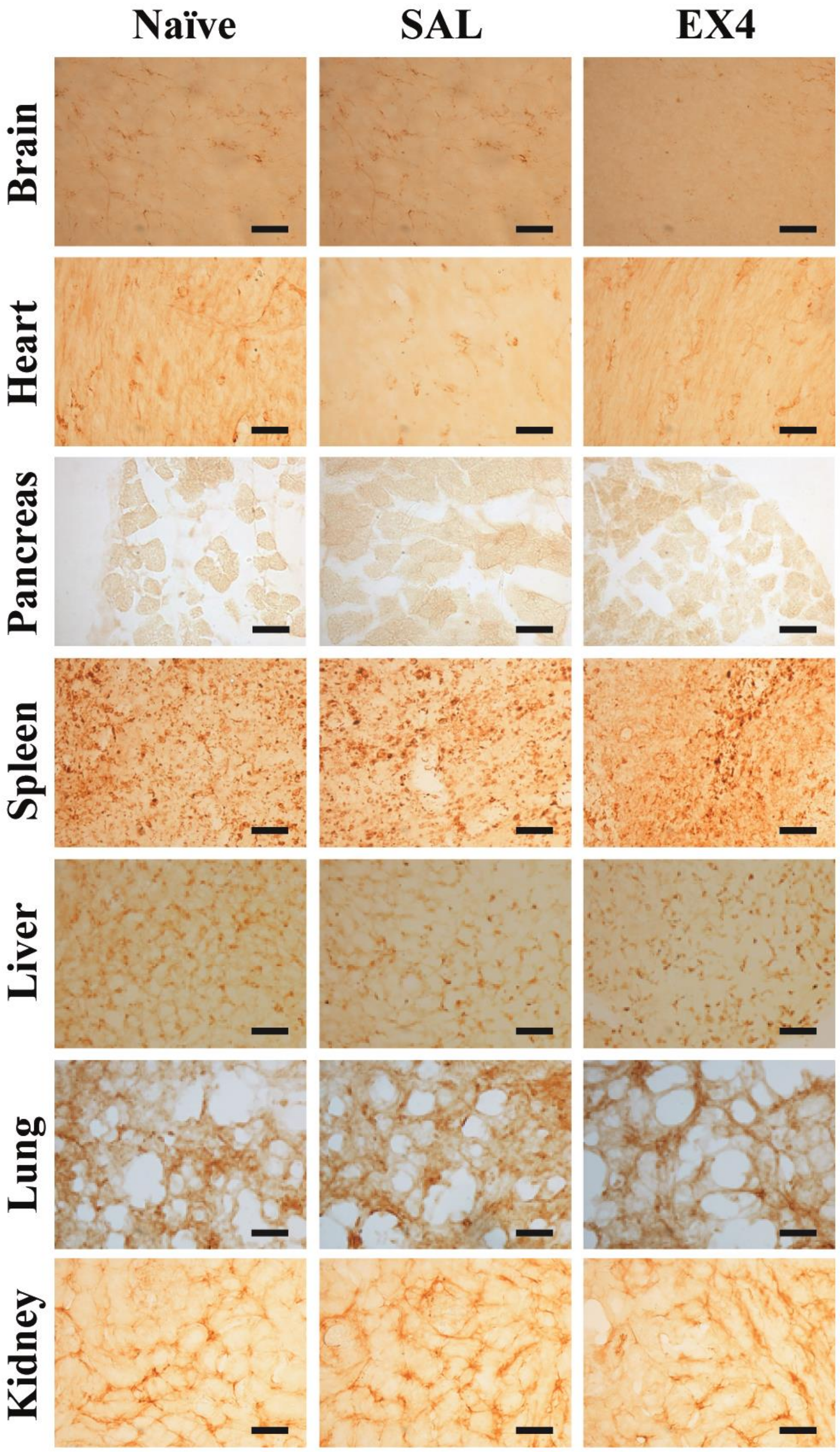

Supplementary figure 6 CD68 micrographs. Representative micrographs of brain, and visceral organs – heart, liver, pancreas, spleen, lung and kidney. Sections were stained with the CD68 macrophage marker. Scale bar = 100µm.

Table 1. Figures datasets

Figure 2 *GLP-1R*										Supplementary Figure 2 *GADPH*								
					**P7**									**P7**				
	**WB**	**CTX**	**HIP**	**HPT**	**THL**	**CBL**	**OB**	**Pons**	**MDL**	**WB**	**CTX**	**HIP**	**HPT**	**THL**	**CBL**	**OB**	**Pons**	**MDL**
**1**	0,9327	0,972	1,116	0,605	0,952	1,752	0,979	0,064	0,163	18,4	18,64	19,48	18,99	18,45	17,8	18,42	18,21	17,6
**2**	0,7859	0,983	1,101	0,608	0,676	1,073	1,167	0,061	0,134	18,35	19,19	19,48	18,36	17,6	17,65	18,54	18,48	17,36
**3**	0,978	1,046	1,049	0,464	0,808	1,899	1,371	0,076	0,122	18,59	19,14	17,16	19,14	17,45	18,5	19,97	19,72	17,14
**4**	0,9438	0,715	2,074	0,584	0,718	1,677	1,156	0,043	0,079	19,13	19,71	21,96	17,45	19,36	18,62	19,25	19,05	19,2
**5**	1,1078	1,064	1,966	0,897	0,618	2,098	1,153	0,044	0,076	19,32	20,49	22,34	17,67	18,63	18,8	18,92	19,59	18,85
**6**	0,7329	1,315	0,747	0,489	0,468	0,858	1,158	0,049	0,072	19,6	20,78	19,94	18,38	18,99	18,71	19,38	19,78	17,64
					**P10**									**P10**				
	**WB**	**CTX**	**HIP**	**HPT**	**THL**	**CBL**	**OB**	**Pons**	**MDL**	**WB**	**CTX**	**HIP**	**HPT**	**THL**	**CBL**	**OB**	**Pons**	**MDL**
**1**	0,74163	1,448	1,889	0,109	0,162	1,324	0,84	0,092	0,069	19,82	22,73	22,6	18,56	18,78	20,66	17,78	17,58	16,08
**2**	0,71513	1,19	1,527	0,126	0,166	1,607	0,917	0,115	0,073	19,72	21,59	22,48	18,99	18,65	20,95	18,02	18,02	15,97
**3**	0,80763	0,917	2,326	0,123	0,249	1,641	1,005	0,104	0,096	20,39	21,22	23,63	19,21	19,39	22,47	18,84	18,57	16,87
**4**	0,73088	1,285	1,061	0,403	0,141	1,377	0,864	0,308	0,408	19,51	21,42	18,84	20,45	20,72	17,53	19,93	18,05	19,2
**5**	0,75725	0,697	1,106	0,325	0,468	1,921	0,752	0,51	0,279	19,78	20,88	19,15	20,67	19,36	18,16	20,14	18,59	21,85
**6**	0,96163	1,218	1,507	0,397	0,618	1,899	1,336	0,428	0,29	18,99	16,37	19,9	21,38	18,99	19,36	20,78	18,78	18,64
					**Adult**									**Adult**				
	**WB**	**CTX**	**HIP**	**HPT**	**THL**	**CBL**	**OB**	**Pons**	**MDL**	**WB**	**CTX**	**HIP**	**HPT**	**THL**	**CBL**	**OB**	**Pons**	**MDL**
**1**	1,091	0,961	0,437	0,074	0,29	3,129	0,603	1,049	0,277	17,04	17,82	16,46	14,35	16,9	17,19	16,2	19,56	16,87
**2**	1,108	0,963	0,437	0,234	0,593	3,247	0,243	0,872	0,28	19,18	20,87	22,49	16,19	19,02	20,3	16,15	19,02	16,57
**3**	1,413	1,051	0,398	0,436	1,159	3,952	0,714	1,175	0,238	19,70	20,94	22,46	17,91	19,64	20,32	18,17	19,63	16,64
**4**	1,244	0,988	0,517	0,303	0,741	2,762	1,496	1,425	0,46	21,70	21,73	19,81	22,03	25,36	19,57	24,26	20,12	22,81
**5**	1,292	0,9	0,415	0,423	1,299	2,478	1,907	1,083	1,038	23,67	23,06	19,35	22,83	25,81	23,87	24,6	24,63	25,64
**6**	1,136	1,008	0,537	0,396	1,273	1,957	1,258	1,113	0,857	23,33	23,62	20,03	22,66	25,9	22,1	24,44	24,5	24,28

Figure 3 *Saline*	*1 high-dose exendin-4*	*4 high-doses exendin-4*	*4 high-doses - 2h delay*	*4 low-doses*

	CTX	PYR	HIP	STR	THL	Overall	CTX	PYR	HIP	STR	THL	Overall	CTX	PYR	HIP	STR	THL	Overall	CTX	PYR	HIP	STR	THL	Overall	Overall
**1**	59	23	40	69	-8	49	6	-3	31	-10	91	13	-3	-3	3	2	-5	-1	6	0	-5	-9	-3	1	10
**2**	-6	-9	51	-5	8	-12	75	100	88	100	64	82	33	2	8	17	6	25	3	1	11	-9	0	0	3
**3**	47	16	51	81	5	48	83	100	93	100	78	87	0	0	0	0	0	0	76	100	93	100	47	77	45
**4**	46	9	52	78	10	45	42	-1	39	7	8	42	0	0	0	0	0	0	31	4	92	15	3	28	59
**5**	13	10	88	63	78	48	7	-15	26	3	-18	-1	-2	-2	3	0	4	-1	78	52	58	71	8	58	4
**6**	53	68	79	20	83	16	4	-10	5	-3	6	-2	0	-1	0	-2	-2	0	-5	-4	92	8	12	-7	0
**7**	72	100	90	64	42	57	-4	-9	-6	-11	-12	-10	0	-4	-1	-1	1	-1	-4	2	6	-2	-2	-2	15
**8**	62	100	64	96	31	78	0	-6	0	0	-11	-3	7	-1	-4	-3	-1	2	-1	-2	-12	-4	-2	-2	67
**9**	64	39	88	94	78	76	0	0	4	0	-7	-3	1	1	5	0	0	0	-2	3	-16	3	-15	-3	64
**19**	46	36	90	55	42	59	37	-7	49	9	3	23	0	0	0	0	0	0	0	-4	7	-4	0	-1	8
**11**	72	100		57		46	-4	-9	-6	-11	-12	-10	4	0	1	2	0	3	-2	1	3	-12	-4	-3	73
**12**	64	39		96		78	0	-6	0	0	-11	-3	4	1	1	2	1	3	6	-3	5	-10	-5	1	52
**13**				55		59	0	0	4	0	-7	-3	0	0	0	0	0	0	3	7	-5	6	-2	2	57
**14**							37	-7	49	9	3	23	2	0	0	0	0	0	11	-2	4	-2	0	5	65

Figure 3 ***Body weight***

	Saline					4 high-doses exendin-4					4 high-doses exendin-4 – 2h					1 high-dose exendin-4					4 low-doses exendin-4				
	**0**	**12**	**24**	**36**	**48**	**0**	**12**	**24**	**36**	**48**	**2**	**14**	**26**	**38**	**48**	**0**	**12**	**24**	**36**	**48**	**0**	**12**	**24**	**36**	**48**
**1**	6,4	6,9	7,3	7,6	7,9	5,7	5,6	5,8	5,9	6,3	6,6	6	5,4	5,3	5,2	5,4	5	5,2	5	5,6	5,7	5,9	6,2	6,5	6,8
**2**	5,4	5,8	6,2	6,5	6,8	6,1	6,3	5,3	6,2	6,5	6,4	5,8	5,2	5,5	5,8	5,8	5,3	5	5	6,2	6,3	6,3	6,2	6,3	6,4
**3**	5,6	6,1	6,6	6,9	7,2	6	5,3	5	5,9	6,1	6,5	6,3	6,1	6,6	7	5,9	5,6	5,6	5,7	5,9	5,7	6,2	6,7	7	7,3
**4**	5,5	5,3	5,1	5,4	5,7	6,1	4,5	5,7	5,7	5,9	5,6	5,7	5,9	5,3	4,8	5,7	5,4	5,4	5,5	5,2	5,9	6	6,2	6,5	6,8
**5**	5,8	6,5	6,8	7,1	7,6	5,6	6,5	4,9	6,1	6,4	6,2	5,6	5,1	5,4	5,6	5,6	5,3	4,9	5,4	5	6,3	6,6	6,9	7,2	7,5
**6**	5,7	6	6,3	6,6	7	6,6	4,4	5,3	5,5	5,8	6,5	6,1	5,6	5,7	5,7	5,5	5,5	5,3	5,6	5,7	5,7	5,6	5,6	5,7	5,8
**7**	6,2	6,1	6,4	6,7	7	5,6	5,1	5,4	5,6	5,9	5,6	6	6,3	6,3	6,4	5,6	5,2	5,4	5,4	5,7	6,1	6,1	6,1	6,2	6,3
**8**	5,6	5,9	5,6	5,7	5,8	5,7	5,1	5,1	5,7	6	6,4	6,9	7,3	7,6	7,9	5,9	5,3	5,2	5,2	5,4	5,5	5,9	6,1	6,3	6,5
**9**	5,4	5,9	6,2	6,2	6,3	5,9	5,7	5,1	5,9	6,1	5,4	5,8	6,2	6,5	6,8	5,8	6,2	6,5	6,7	6,8	5,8	6,2	6,4	6,6	6,8
**10**	5,1	5,2	5	5,3	5,7	5,4	5	5,7	5,2	5,4	5,6	6,1	6,6	6,9	7,2	5,7	6,1	6,3	6,5	6,6	6,2	6,5	6,7	6,9	7,1

**11**	5,2	5,7	6,2	6,8	7,4	5,8	5,6	5,6	5,8	6	5,5	5,3	5,1	5,4	5,7	5,5	6	6,3	6,4	6,5	5,6	5,9	6,1	6,3	6,5
**12**	5,6	5,7	6,2	6,7	7,2	5,9	5,5	5,4	5,7	5,9	5,2	5,2	5,1	5,2	5,2	5,9	6	6,5	6,7	6,9	5,6	6	6,2	6,4	6,6
**13**	5,4	5,9	6,5	7,1	7,7	5,7	5,3	5,3	5,5	5,7	5,7	5,4	5,2	5,7	6,1	6	5,4	5	4,9	4,7	5	5,3	5,5	5,7	5,9
**14**	5,7	6,1	6,3	6,6	6,8	5,6	5,3	5,5	5,5	5,7	5,6	5,3	5,1	5,7	6,3	5,5	6,1	6,3	6,4	6,5	4,9	5,3	5,5	5,7	5,9

Figure 4 ***TUNEL***

	**Saline**							**4 high-doses exendin-4**							**4 high-doses exendin-4 - 2h delay**						
	**CTX**	**PYR**	**EXC**	**HIP**	**STR**	**THL**	**Overall**	**CTX**	**PYR**	**EXC**	**HIP**	**STR**	**THL**	**Overall**	**CTX**	**PYR**	**EXC**	**HIP**	**STR**	**THL**	**Overall**
**1**	409,4	17,5	90,0	0,0	112,0	0,0	104,8	0,0	0,0	0,0	111,7	0,0	0,0	18,6	0,0	0,0	0,0	0,0	0,0	0,0	0,0
**2**	72,5	0,0	16,5	0,0	543,3	16,7	108,2	220,0	5,0	90,0	212,5	411,7	0,0	156,5	0,0	0,0	0,0	0,0	0,0	0,0	0,0
**3**	841,7	124,0	95,0	329,2	808,6	21,7	370,0	0,0	0,0	0,0	81,7	0,0	0,0	13,6	55,0	200,0	17,7	58,3	250,0	23,3	100,7
**4**	1,1	0,0	0,1	62,1	1,1	0,0	10,7	0,0	0,0	0,0	0,0	0,0	0,0	0,0	67,9	0,8	22,5	133,3	43,9	3,3	45,3
**5**	301,8	13,8	29,4	262,0	153,8	5,3	127,7	0,0	0,0	0,0	74,2	0,0	0,0	12,4	123,3	0,2	3,8	23,3	7,5	2,2	26,7
**6**	719,7	260,5	198,5	357,8	735,4	422,2	449,0	0,0	0,0	0,0	0,0	0,0	0,0	0,0	0,0	0,0	0,0	0,0	0,0	0,0	0,0
**7**	530,4	357,1	55,0	362,5	722,7	354,2	397,0	0,0	0,0	0,0	13,3	0,0	0,0	2,2	0,0	0,0	0,0	20,8	0,0	0,0	3,5
**8**	750,0	262,5	153,1	355,6	634,6	568,9	454,1	0,0	0,0	0,0	135,0	0,0	0,0	22,5	0,0	0,0	0,0	0,0	0,0	0,0	0,0
**9**	786,7	277,5	100,5	380,6	551,8	312,2	401,5	4,6	0,6	0,8	4,2	1,8	0,0	2,0	0,0	0,0	0,0	13,3	0,0	0,0	2,2
**10**	828,3	352,5	162,0	441,7	716,1	420,8	486,9	0,6	0,0	0,0	0,0	0,0	0,0	0,1	0,0	0,0	0,0	0,0	0,0	0,0	0,0
**11**	409,4	17,5	90,0	0,0	112,0	0,0	104,8	73,3	6,2	10,1	23,3	21,1	1,7	22,6	0,0	0,0	0,0	0,0	0,0	0,0	0,0
**12**	719,7	260,5	198,5	357,8	735,4	422,2	449,0	0,0	0,0	0,0	0,0	0,0	0,0	0,0	0,0	0,0	0,0	0,8	0,0	0,0	0,1
**13**	828,3	352,5	162,0	441,7	716,1	420,8	486,9	0,0	0,0	0,0	0,0	0,0	0,0	0,0	0,0	0,0	0,0	3,5	0,0	0,0	0,6
**14**	530,4	357,1	55,0	362,5	722,7	354,2	397,0	0,0	0,0	0,0	0,0	0,0	0,0	0,0	0,0	0,0	0,0	0,8	0,0	0,0	0,1

Figure 5 ***Microglia activation***

	**Saline**							**4 high-doses exendin-4**							**4 high-doses exendin-4 - 2h delay**						
	**CTX**	**PYR**	**HIP**	**STR**	**THL**	**EXC**	**Overall**	**CTX**	**PYR**	**HIP**	**STR**	**THL**	**EXC**	**Overall**	**CTX**	**PYR**	**HIP**	**STR**	**THL**	**EXC**	**Overall**

**1**	4,0	1,8	4,0	4,0	4,0	4,0	4,0	0,0	0,0	0,0	0,0	0,0	0,0	0,4	0,3	0,0	2,0	0,0	0,0	0,3	0,4
**2**	0,2	0,0	1,5	0,8	0,8	0,8	0,8	0,5	0,5	0,5	0,5	0,5	0,5	2,9	0,0	0,0	0,0	0,0	0,0	0,0	0,0
**3**	4,0	3,4	4,0	4,0	4,0	4,0	4,0	0,0	0,0	0,0	0,0	0,0	0,0	0,1	4,0	4,0	4,0	4,0	4,0	4,0	4,0
**4**	4,0	1,7	2,5	3,4	3,4	3,4	3,4	0,0	0,0	0,0	0,0	0,0	0,0	0,1	4,0	0,0	2,0	0,3	0,0	2,3	1,4
**5**	4,0	2,4	3,5	4,0	1,5	4,0	3,2	0,0	0,0	0,0	0,0	0,0	0,0	0,2	4,0	4,0	2,0	4,0	1,0	4,0	3,2
**6**	4,0	1,9	3,5	2,6	0,8	4,0	2,8	0,0	0,0	0,0	0,0	0,0	0,0	0,2	0,0	0,0	0,0	0,0	0,0	0,0	0,0
**7**	4,0	4,0	4,0	4,0	4,0	4,0	4,0	0,0	0,0	1,0	0,0	0,3	0,5	0,3	0,0	0,0	0,0	0,0	0,0	0,0	0,0
**8**	4,0	4,0	4,0	4,0	4,0	4,0	4,0	0,4	0,0	1,8	0,0	0,7	1,8	0,7	0,0	0,0	0,0	0,0	0,0	0,0	0,0
**9**	4,0	4,0	4,0	4,0	4,0	4,0	4,0	0,0	0,0	0,0	0,0	0,0	0,0	0,4	0,5	0,0	1,5	0,3	0,5	1,0	0,6
**10**	4,0	3,0	4,0	4,0	4,0	4,0	3,8	0,5	0,5	0,5	0,5	0,5	0,5	2,9	0,0	0,0	0,0	0,0	0,0	0,0	0,0
**11**	4,0	1,8	4,0	4,0	4,0	4,0	4,0	0,0	0,0	0,0	0,0	0,0	0,0	0,1	0,0	0,0	0,0	0,0	0,0	0,0	0,0
**12**	0,2	0,0	1,5	0,8	0,8	0,8	0,8	0,0	0,0	0,0	0,0	0,0	0,0	0,1	0,0	0,0	0,0	0,0	0,0	0,0	0,0
**13**	4,0	3,4	4,0	4,0	4,0	4,0	4,0	0,0	0,0	0,0	0,0	0,0	0,0	0,2	0,0	0,0	0,0	0,0	0,0	0,0	0,0
**14**	4,0	1,7	2,5	3,4	3,4	3,4	3,4	0,0	0,0	0,0	0,0	0,0	0,0	0,2	0,7	0,0	1,0	0,0	0,0	0,7	0,4

Figure 5 ***Astrogliosis***

	**Saline**							**4 high-doses exendin-4**							**4 high-doses exendin-4 - 2h delay**						
	**CTX**	**PYR**	**HIP**	**STR**	**THL**	**EXC**	**Overall**	**CTX**	**PYR**	**HIP**	**STR**	**THL**	**EXC**	**Overall**	**CTX**	**PYR**	**HIP**	**STR**	**THL**	**EXC**	**Overall**
**1**	5,3	2,0	5,3	1,3	0,8	3,7	3,1	0,7	3,3	0,1	0,1	0,0	0,1	0,7	0,7	3,3	0,1	0,1	0,0	0,1	0,7
**2**	5,2	1,9	16,4	7,8	9,7	8,4	8,2	0,8	1,5	0,5	0,1	0,7	0,4	0,7	0,8	1,5	0,5	0,1	0,7	0,4	0,7
**3**	11,1	11,1	4,8	3,6	0,4	5,8	6,1	4,2	2,1	0,2	0,7	0,9	1,1	1,5	12,2	2,1	0,6	0,7	10,8	0,1	5,9
**4**	2,5	3,1	3,3	0,2	0,7	1,4	1,9	4,5	2,0	1,8	2,6	0,4	2,8	2,4	4,2	2,0	0,2	2,6	0,9	1,1	1,5
**5**	6,0	9,3	5,4	1,7	0,1	4,2	4,5	2,6	4,9	2,2	0,8	0,8	0,8	2,0	4,5	11,4	1,8	4,1	0,4	2,8	2,4
**6**	7,6	7,6	9,5	10,2	3,3	4,1	7,1	1,1	1,2	6,4	4,9	1,4	1,1	2,7	9,8	4,9	26,4	0,8	5,4	12,2	11,5
**7**	3,5	3,5	5,5	5,5	2,5	5,5	4,3	1,7	3,4	1,2	0,4	5,5	0,6	2,1	2,6	1,2	2,2	4,9	0,8	0,8	2,0
**8**	10,0	10,0	9,2	9,2	2,5	5,5	7,7	1,9	1,9	1,9	1,9	1,9	1,9	1,9	1,1	3,4	6,4	0,4	1,4	1,1	2,7
**9**	6,4	6,4	6,8	6,8	3,6	9,8	6,6	1,2	1,2	1,2	1,2	1,2	1,2	1,2	1,7	1,9	1,2	1,9	5,5	0,6	2,1
**10**	22,2	22,2	22,2	22,2	22,2	22,2	22,2	10,6	13,6	3,2	17,7	1,1	2,6	8,1	1,9	1,2	1,9	1,2	1,9	1,9	1,9

**11**	12,2	12,2	6,0	6,0	10,8	10,1	9,5	0,7	0,7	0,7	0,7	0,7	0,7	0,7	1,2	13,6	1,2	17,7	1,2	1,2	1,2
**12**	9,8	11,4	26,4	4,1	5,4	12,2	11,5	21,5	12,9	2,4	1,9	2,2	3,7	7,4	10,6	0,7	3,2	0,7	1,1	2,6	8,1
**13**	10,6	13,6	3,2	17,7	1,1	2,6	8,1								0,7	12,9	0,7	1,9	0,7	0,7	0,7
**14**	21,5	12,9	2,4	1,9	2,2	3,7	7,4								21,5		2,4		2,2	3,7	7,4

Figure 5 *Endothelial activation*

	**Saline**							**4 high-doses exendin-4**							**4 high-doses exendin-4 - 2h delay**						
	**CTX**	**PYR**	**HIP**	**STR**	**THL**	**EXC**	**Overall**	**CTX**	**PYR**	**HIP**	**STR**	**THL**	**EXC**	**Overall**	**CTX**	**PYR**	**HIP**	**STR**	**THL**	**EXC**	**Overall**
**1**	15,8	15,9	16,0	16,0	16,0	16,0	16,0	0,6	0,7	0,5	0,3	0,4	0,5	0,5	0,6	0,7	0,5	0,3	0,4	0,5	0,5
**2**	2,0	0,5	0,5	0,5	0,5	0,5	0,5	1,2	3,5	3,7	1,2	1,3	1,4	2,0	1,2	3,5	3,7	1,2	1,3	1,4	2,0
**3**	4,2	4,0	4,0	4,0	4,0	4,0	4,0	0,9	2,5	1,5	3,1	1,2	1,4	1,8	0,9	2,5	1,5	3,1	1,2	1,4	1,8
**4**	1,2	1,3	1,2	1,2	1,2	1,2	1,2	1,1	0,8	0,8	0,5	0,5	0,7	0,7	1,1	0,8	0,8	0,5	0,5	0,7	0,7
**5**	2,2	2,2	1,5	9,9	7,8	4,0	4,6	0,8	0,6	0,8	0,8	1,4	0,6	0,8	0,8	0,6	0,8	0,8	1,4	0,6	0,8
**6**	1,4	1,0	1,5	0,4	0,3	0,9	0,9	0,6	0,5	0,5	0,5	0,4	0,5	0,5	0,6	0,5	0,5	0,5	0,4	0,5	0,5
**7**	2,0	2,0	2,0	2,1	2,1	2,1	2,1	0,6	0,7	0,5	0,3	0,4	0,5	0,5	1,2	3,5	3,7	1,2	1,3	1,4	2,0
**8**	9,6	9,6	9,6	9,6	9,6	9,6	9,6	1,2	3,5	3,7	1,2	1,3	1,4	2,0	2,0	2,0	2,0	2,1	2,1	2,1	2,1
**9**	1,0	1,7	0,9	3,5	1,3	10,5	3,2	0,9	2,5	1,5	3,1	1,2	1,4	1,8	1,2	3,5	3,7	1,2	1,3	1,4	2,0
**10**	4,2	4,0	4,0	5,2	4,6	5,3	4,5	1,1	0,8	0,8	0,5	0,5	0,7	0,7	0,9	2,5	1,5	3,1	1,2	1,4	1,8
**11**	2,0	2,0	2,0	2,1	2,1	2,1	2,1	0,8	0,6	0,8	0,8	1,4	0,6	0,8	0,6	0,5	0,5	0,5	0,4	0,5	0,5
**12**	2,0	0,5	0,5	0,5	0,5	0,5	0,5	0,6	0,5	0,5	0,5	0,4	0,5	0,5	2,0	2,0	2,0	2,1	2,1	2,1	2,1
**13**	4,2	4,0	4,0	4,0	4,0	4,0	4,0	1,2							0,8	0,6	0,8	0,8	1,4	0,6	0,8
**14**	9,6	9,6	9,6	9,6	9,6	9,6	9,6	0,9							0,6	0,5	0,5	0,5	0,4	0,5	0,5

Figure 6 *Glucose* / *Body weight*

	Glucose					*Body weight*														
	**Naïve**	**Exendin-4**				**Naïve**					**Saline**					**Exendin-4**				
		**0.5h**	**1h**	**2h**	**4h**	**0h**	**12h**	**24h**	**36h**	**48h**	**0h**	**12h**	**24h**	**36h**	**48h**	**0h**	**12h**	**24h**	**36h**	**48h**
**1**	4,8	5,8	6,1	6,1	6,5	6,7	7,2	8,1	8,4	8,9	6,6	6,9	7,4	7,9	8,1	6,6	5,8	5,7	5,8	6,3

**2**	6,8	7,0	7,5	5,3	5,6	6,7	7,2	6,7	7,1	7,3	6,1	6,7	7,3	7,8	8,0	6,8	6,4	6,5	6,7	7,1
**3**	4,0	6,6	4,7	5,6	7,7	5,7	6,4	8,0	8,3	8,4	6,8	6,8	7,7	8,2	8,3	6,2	5,8	5,9	6,0	6,4
**4**	7,2	6,7	6,1	5,5		6,0	5,9	7,0	7,3	7,7	6,2	7,0	7,5	8,0	8,4	6,0	6,2	5,7	5,9	6,4

Figure 6 ***Blood counts***

	**Total WBC**			**Neutrophils**			**Lymphocytes**			**Monocytes**			**Eosinophils**			**Basophils**		
	**Naïve**	**SAL**	**EX4**	**Naïve**	**SAL**	**EX4**	**Naïve**	**SAL**	**EX4**	**Naïve**	**SAL**	**EX4**	**Naïve**	**SAL**	**EX4**	**Naïve**	**SAL**	**EX4**
**1**	10,8	5,5	13,6	0,6	0,4	1,4	9,2	4,4	11,0	0,2	0,2	0,2	0,0	0,0	0,1	0,0	0,0	0,1
**2**	9,9	6,9	6,1	0,8	0,8	2,4	8,8	5,4	3,0	0,2	0,2	0,2	0,0	0,0	0,0	0,0	0,0	0,0
**3**	10,8	13,7	5,3	0,4	1,0	0,6	9,8	10,8	4,0	0,2	0,2	0,2	0,0	0,0	0,0	0,0	0,1	0,0
**4**	8,2	11,4	9,1	0,4	3,2	1,6	7,4	6,8	6,4	0,2	0,6	0,4	0,0	0,0	0,0	0,0	0,0	0,1
**5**	18,9	33,3	11,1	1,4	2,6	2,8	14,2	27,6	7,4	0,6	0,6	0,8	0,1	0,0	0,0	0,0	0,0	0,0
**6**		6,3	10,8		0,6	0,8		4,4	8,2			0,4			0,0			0,0

	**HCT**			**Platelets**			**RBC**			**Haemoglobin**			**MCV**		
	**Naïve**	**SAL**	**EX4**	**Naïve**	**SAL**	**EX4**	**Naïve**	**SAL**	**EX4**	**Naïve**	**SAL**	**EX4**	**Naïve**	**SAL**	**EX4**
**1**	57,0	50,0	61,0	1740,0	1340,0	1560,0	16,2	13,6	17,8	21,0	15,0	20,0	70,8	72,8	68,8
**2**	31,0	30,0	50,0	780,0	1440,0	1660,0	9,6	8,8	15,0	10,0	13,0	16,0	63,8	68,8	66,5
**3**	38,0	56,0	60,0	900,0	840,0	1460,0	11,4	16,4	18,2	12,0	18,0	19,0	66,5	69,1	66,1
**4**	40,0	41,0	62,0	2200,0	1380,0	1900,0	11,6	12,0	18,2	10,0	14,0	19,0	68,7	68,4	68,1
**5**	82,0	65,0	41,0	1500,0	1660,0	1560,0	22,8	17,8	24,0	25,0	22,0	27,0	71,2	73,4	68,5
**6**		45,0	46,0		1340,0	1340,0		12,4	27,8		13,0	29,0		73,2	66,2

Figure 7 ***CD68 threshold averages***

	**Brain**			**Heart**			**Pancreas**			**Spleen**			**Liver**			**Lung**			**Kidney**		
	**Naïve**	**SAL**	**EX4**	**Naïve**	**SAL**	**EX4**	**Naïve**	**SAL**	**EX4**	**Naïve**	**SAL**	**EX4**	**Naïve**	**SAL**	**EX4**	**Naïve**	**SAL**	**EX4**	**Naïve**	**SAL**	**EX4**
**1**	0,32	0,74	0,17	1,22	1,01	1,61	0,36	0,23	0,58	13,26	9,90	4,74	10,77	8,87	8,88	24,04	25,46	27,49	23,29	14,52	14,48
**2**	0,70	1,04	0,30	1,08	1,16	1,15	0,40	0,15	0,11	13,40	9,33	7,04	12,65	6,87	10,39	27,82	26,74	27,89	21,82	18,48	22,76
**3**	0,20	0,60	0,37	1,52	2,09	1,03	0,36	0,15	0,09	9,51	8,04	11,20	7,035	10,87	12,30	23,78	26,67	18,96	21,53	31,30	22,13

**4**	0,69	0,46	1,10	1,76	1,95	1,03	0,21	0,12	0,20	13,88	7,71	13,32	9,41	10,41	10,32	25,00	25,64	28,86	15,89	28,58	21,56
**5**	0,60	0,94	1,07	1,47	1,12	1,63	0,15	0,27	0,14	11,62	8,92	17,14	13,96	9,198	9,75	23,16	29,13	29,92	19,07	19,57	15,06
**6**		0,72	0,22		1,47	1,78		0,17	0,08		7,90	9,19		7,63	10,52		27,98	28,59		17,78	18,35

Figure 8 *Macroscopic score*					***Body weight***																
	**NT SAL**	**NT EX4**	**HT SAL**	**HT EX4**	**NT SAL**				**NT EX4**				**HT SAL**				**HT EX4**			
					**P10**	**P12**	**P14**	**P17**	**P10**	**P12**	**P14**	**P17**	**P10**	**P12**	**P14**	**P17**	**P10**	**P12**	**P14**	**P17**
**1**	1,5	0,0	3,0	0,0	5,6	6,8	7,8	8,2	5,6	5,9	7,5	7,6	7,1	8,2	8,6	8,6	6,3	7,1	7,2	7,5
**2**	2,0	2,0	2,0	0,0	6,8	8,6	7,9	9,0	5,4	5,8	6,9	7,1	6,9	7,1	8,0	8,5	6,2	6,9	7,3	7,6
**3**	2,5	2,0	2,0	0,0	6,6	5,7	7,1	7,5	6,1	6,5	7,8	7,9	7,5	8,8	10,2	11,0	6,5	7,2	7,3	7,8
**4**	2,5	0,0	3,0	2,0	5,7	7,0	8,1	8,4	6,0	7,1	7,0	8,7	6,1	7,1	8,4	8,9	6,2	6,2	6,9	7,5
**5**	3,0	2,0	2,0	0,0	5,7	6,5	6,9	7,5	6,5	7,7	7,4	9,1	6,5	8,2	9,2	9,8	7,0	8,6	8,9	9,8
**6**	2,0	0,0	0,0	0,0	5,8	7,7	8,3	9,8	7,1	8,0	9,1	9,8	6,9	7,6	7,9	8,9	7,0	8,1	9,1	10,4
**7**	3,0	3,0	2,0	0,0	6,2	5,6	5,5	4,4	6,6	6,1	6,5	6,8	7,8	8,3	9,1	9,8	6,3	8,1	8,8	9,4
**8**	3,0	0,0	3,0	1,0	6,1	6,2	6,5	7,1	7,9	7,8	8,0	8,9	6,5	6,8	7,0	6,9	6,8	7,3	8,3	8,9
**9**	2,0	0,0	2,0	0,0	6,2	6,3	7,2	7,6	6,7	7,5	7,8	7,8	6,7	6,9	7,7	8,5	7,2	8,1	8,9	9,2
**10**	3,0	0,0	3,0	1,0	7,0	8,2	8,6	9,0	7,9	8,1	9,1	10,8	6,2	5,4	5,6	5,6	7,2	7,5	8,6	9,3
**11**	2,0	1,0	2,0	0,0	6,3	7,4	7,7	8,3	6,6	7,0	7,4	9,0	6,4	7,5	7,1	7,3	6,7	7,3	8,3	8,9
**12**	3,0	2,0	0,0	0,0	6,3	7,3	7,6	7,6	6,5	6,9	7,1	7,9	7,0	8,1	8,9	9,4	6,7	7,3	8,3	8,9
**13**	2,0	3,0	0,0	0,0	7,2	9,0	8,7	9,6	7,4	8,1	8,6	9,6	7,7	9,8	10,0	10,9	6,9	7,4	8,3	9,1
**14**	3,0	1,5	3,0	2,0	6,9	7,1	7,2	7,4	6,2	7,1	7,2	8,5	5,4	6,3	7,0	7,5	6,5	7,2	7,7	7,6
**15**	3,0	2,0	0,0	0,0	6,5	6,3	6,5	5,9	6,7	7,8	8,2	8,9	6,2	8,2	8,4	9,2	6,5	8,1	8,2	8,6
**16**	3,0	0,0	0,0	0,0	6,9	7,2	7,5	7,9	6,5	6,7	7,6	8,5	6,5	7,3	7,9	8,8	7,3	8,4	9,0	10,1
**17**	0,0	2,0	0,0	0,0	6,7	7,0	7,1	7,6	6,2	6,3	7,3	7,9	6,2	6,5	8,0	8,8	6,2	6,7	7,6	8,5
**18**	3,0	1,5	3,0	3,0	6,8	7,2	7,4	7,7	6,0	6,2	7,4	8,1	6,0	6,5	7,5	7,9	6,5	7,1	7,5	8,6
**19**	2,0	0,0	1,5	0,0	6,6	6,8	7,5	8,0	6,5	6,9	7,9	8,6	6,3	6,9	7,7	8,5	6,0	7,2	8,3	9,0
**20**	3,0	3,0	0,0	0,0	6,9	7,1	7,4	7,9	6,0	6,3	7,2	8,0	6,5	6,9	8,1	9,1	6,0	7,1	7,9	8,6
**21**	3,0	0,0	0,0	1,0	4,7	5,7	6,7	7,7	6,1	6,5	7,5	8,4	6,7	7,2	8,0	8,9	6,3	6,9	7,7	8,8
**22**	2,5	0,0	2,5	0,0	5,4	5,6	6,8	7,9	5,7	6,7	7,8	8,9	4,5	5,3	6,1	6,8	6,5	6,7	8,0	8,9
**23**	3,0	0,0	0,0	0,0	5,4	6,4	7,4	8,4	4,9	5,5	6,3	6,8	5,3	6,4	7,6	8,8	5,7	6,9	7,9	8,6
**24**	1,0	0,0	0,0	0,0	4,0	5,2	6,4	7,6	5,0	5,8	6,9	7,5	5,0	6,3	7,6	7,9	5,1	5,8	6,8	7,4

**25**		0,0	0,0	0,0					4,9	5,5	6,5	6,9	4,8	5,7	6,6	7,5	5,1	5,5	5,9	6,2

Figure 8 ***MAP2***

	**NT SAL**					**NT EX4**					**HT SAL**					**HT EX4**				
	**L1**	**L2**	**L3**	**L4**	**L5**	**L1**	**L2**	**L3**	**L4**	**L5**	**L1**	**L2**	**L3**	**L4**	**L5**	**L1**	**L2**	**L3**	**L4**	**L5**
**1**	7	14	32	24	51	-2	1	4	-3	-7	42	27	33	55	35	-4	2	1	2	-1
**2**	26	27	39	44	34	2	3	42	49	36	24	23	28	38	33	4	-5	8	0	1
**3**	32	34	25	31	40	4	37	-5	1	0	18	68	68	31	0	-6	-42	-47	-5	0
**4**	18	18	73	73	32	1	1	40	40	40	61	15	35	69	31	-30	3	-8	36	-8
**5**	12	54	19	26	31	47	5	44	-2	-3	8	-2	2	4	51	-1	9	-5	-9	-4
**6**	52	-17	61	68	70	-3	54	4	45	50	2	8	21	26	25	-1	2	-2	3	0
**7**	53	57	42	60	63	0	-5	0	-2	2	22	29	37	47	0	-7	-10	10	-5	0
**8**	35	41	90	41	44	0	0	53	0	0	9	9	8	25	38	17	13	13	18	6
**9**	95	97	30	97	94	0	51	4	57	48	4	0	0	0	-5	12	7	5	13	15
**10**	21	54	64	35	28	0	4	21	31	68	12	31	35	37	-1	-2	2	1	8	8
**11**	29	33	31	74	83	3	8	1	72	6	-1	4	-6	-3	46	-1	-5	3	4	2
**12**	63	65	71	31	71	-1	61	4	1	0	0	0	0	-3	1	-4	-5	2	-4	-1
**13**	38	42	43	73	44	2	5	0	-2	-2	-2	46	50	46	2	5	29	41	4	-1
**14**	19	24	26	39	56	0	-5	0	7	-3	-3	4	-1	0	3	-6	4	3	47	42
**15**	35	44	44	31	49	0	-1	0	0	0	9	2	-13	3	70	-1	0	5	-1	3
**16**	24	29	25	47		3	0	63	0	79	2	0	3	1	52	-2	-5	-2	3	1
**17**	56	36	66	27		1	52	0	66	-4	-9	21	88	88	0	-1	3	29	2	-1
**18**	24	29	31	69		0	4	0	-4	0	4	0	23	23		-1	-2	0	33	31
**19**	18	29	31	32		0	0	-1	0	-2	0	-4	-3	0		-3	0	-2	3	0
**20**	-1	0	27	33			0	3	0	6		29	-3	3		-2	-6	0	2	-1
**21**	0		0	29			-10	-5	-5	5		2	34	-4		-6	4	1	0	0
**22**				1			-5		1			-8	-2	7		-3	0	-4	-1	-1
**23**												4	-4	6			2	0	-1	-1
**24**													1					3	-10	-3
**25**																			0	1

Supplementary figure 1

	Sentinel temperature															
	**NT**								**HT**							
	**1**	**2**	**3**	**4**	**5**	**6**	**7**	**8**	**1**	**2**	**3**	**4**	**5**	**6**	**7**	**8**
**0h**	34,3	34,2	33,7	34,8	34,4	34,8	33,9	33,8	32,5	32,0	32,9	33,5	33,8	33,5	32,0	32,0
**0.1h**	36,4	36,2	35,7	36,2	36,4	35,7	36,0	35,9	33,0	32,5	33,3	32,8	33,0	32,5	32,5	32,5
**0.5h**	35,7	35,9	35,9	36,0	36,1	35,8	36,0	35,9	32,9	32,8	33,0	33,0	33,3	32,7	32,6	32,7
**1h**	36,1	36,1	36,0	36,0	36,2	36,0	36,1	36,2	33,1	33,1	33,2	33,0	32,9	33,2	32,7	32,9
**1.5h**	36,2	36,1	36,1	36,0	36,0	35,9	36,1	36,2	33,1	33,1	33,0	33,1	33,0	33,0	33,0	32,7
**2h**	36,2	36,1	36,1	36,0	36,1	36,1	36,0	36,1	33,3	33,1	33,1	33,1	33,1	33,0	32,9	32,8
**2.5h**	36,2	36,1	36,0	36,0	36,0	36,0	36,0	36,2	33,1	33,1	32,9	33,0	33,0	32,9	33,1	33,0
**3h**	36,2	36,0	36,0	36,1	36,0	36,1	35,9	36,1	33,1	33,1	33,0	33,0	32,9	33,0	33,0	33,0
**3.5h**	36,1	36,0	36,0	36,0	36,0	36,2	36,0	36,1	33,1	33,1	33,1	33,0	32,7	33,0	33,1	32,9
**4h**	36,2	36,1	36,0	36,0	36,2	36,1	36,2	36,1	33,1	33,0	33,0	33,0	33,1	33,0	33,0	32,9
**4.5h**	36,1	36,1	36,0	36,0	36,1	36,1	36,2	36,1	32,9	33,1	33,1	33,0	33,1	32,9	33,0	33,0
**5h**	36,1	36,0	36,1	36,0	36,2	36,1	36,1	36,1	33,0	33,1	33,1	33,0	32,9	33,0	33,0	32,9

	Chamber temperature																***Room temperature***							
	**NT**								**HT**															
	**1**	**2**	**3**	**4**	**5**	**6**	**7**	**8**	**1**	**2**	**3**	**4**	**5**	**6**	**7**	**8**	**1**	**2**	**3**	**4**	**5**	**6**	**7**	**8**
**0h**	37,3	36,0	36,0	36,0	36,0	35,7	36,0	36,0	33,5	32,0	35,5	34,5	35,0	34,5	34,5	33,6	23,1	23,2	23,1	22,9	22,8	23,0	23,0	22,6
**0.1h**	35,6	36,5	34,7	35,5	35,8	35,7	35,5	35,8	35,9	32,4	34,0	32,6	32,0	32,6	32,8	33,0	22,9	23,2	23,0	22,9	22,8	23,0	23,0	22,6
**0.5h**	35,7	35,7	35,6	35,1	35,2	35,7	35,1	35,2	34,8	32,4	33,9	32,4	33,0	32,4	32,5	32,9	23,1	23,2	23,1	22,8	22,8	23,0	23,0	22,6
**1h**	35,	35,1	35,	34,9	34,8	35,3	34,	34,8	33,3	33,0	32,4	32,0	33,	32,0	32,0	32,2	23,1	23,3	23,0	22,9	22,8	22,9	23,0	22,6

	3		3				9						0											
**1.5h**	35,3	35,1	35,3	34,9	34,8	35,3	34,9	34,9	33,1	33,0	32,5	32,4	33,0	32,7	32,4	32,4	23,1	23,3	23,0	22,9	22,8	22,8	23,1	22,7
**2h**	35,3	35,1	35,3	34,9	34,9	35,4	34,9	34,9	33,0	33,0	32,3	32,0	33,0	33,0	32,0	32,0	23,1	23,3	23,0	23,0	22,9	22,8	23,1	22,8
**2.5h**	35,2	35,0	35,2	34,8	34,8	35,2	34,8	34,8	32,9	33,1	32,3	32,0	33,1	33,1	32,0	32,0	23,0	23,4	23,0	23,2	22,9	22,8	23,1	22,8
**3h**	35,2	35,0	35,2	34,9	34,9	35,1	34,9	34,9	33,0	33,0	32,3	32,0	33,0	33,0	32,0	32,0	23,0	23,4	23,1	23,2	22,9	22,8	23,0	22,8
**3.5h**	35,1	35,0	35,3	34,9	34,9	34,9	34,9	34,9	32,4	32,9	32,5	32,0	32,9	32,9	32,0	32,0	23,0	23,3	23,1	23,1	22,9	22,8	23,0	22,9
**4h**	35,2	35,0	35,2	34,9	34,9	34,6	34,7	34,9	32,7	32,9	32,2	32,1	32,9	32,9	32,1	32,1	23,0	23,3	23,1	23,1	22,9	22,7	23,0	22,9
**4.5h**	35,2	35,0	35,2	35,1	35,0	34,4	34,3	35,0	32,9	32,9	32,3	32,0	32,9	32,7	32,0	32,0	23,0	23,3	23,0	23,1	22,8	22,7	23,0	23,0
**5h**	35,1	35,0	35,2	35,2	35,1	34,3	34,0	35,2	32,9	32,9	31,6	32,0	32,9	32,0	32,0	32,0	23,0	23,5	23,0	23,1	22,8	22,7	23,0	23,1

Supplementary figure 4

cAMP

	**Saline**	**Exendin-4**			
	**2h**	**2h**	**4h**	**8h**	**12h**
**1**	1,73	12,38	11,22	7,36	4,87
**2**	2,24	13,5	10,89	4,1	4,76
**3**	2,28	11,55	11,57	7,34	6,38
**4**	1,82	12,51	11,74	8,03	2,35

Abbreviations: 2H - exendin 4, 4 high-doses 2h delay; CBL – cerebellum; CTX – isocortex; DIL – exendin 4, 4 low-doses; EX1 – exendin 4, 1 high-dose; EX4 – exendin 4, 4 high-doses; EXC – external capsule; HCT – hematocrit; HIP – hippocampus; HPT – hypothalamus; HT – hypothermia; MCV – mean corpuscular volume; MDL – medulla; NT – normothermia; OB – olfactory bulbs; PYR – pyriform cortex; RBC – red blood cells; THL – thalamus; SAL – saline; STR – striatum; WB – whole brain; WBC – white blood cells.

Table 2. Statistical comparisons

Figure 3 + Suppl. Fig. 3	*Tissue infarction*						*HI body weights*				
**Analysis**	**Overall**	**CTX**	**PYR**	**HIP**	**STR**	**THL**	**0h**	**12h**	**24h**	**36h**	**48h**
**Kruskal-Wallis test *P* value**	**0,0007**	**0,0006**	**0,0026**	**0,0013**	**0,0006**	**0,0366**	0,2901	**0,0108**	**0,0017**	**0,0018**	**0,0019**
**Dunn's multiple comparisons test**	*P* Value	*P* Value	*P* Value	*P* Value	*P* Value	*P* Value	*P* Value	*P* Value	*P* Value	*P* Value	*P* Value
**SAL vs. EX1**	**0,0279**	0,7208	**0,0013**	0,1886	**0,0468**	0,0648	>0,9999	>0,9999	0,2766	**0,0212**	**0,0148**
**SAL vs. EX4**	**0,0214**	**0,0222**	**0,0629**	**0,0005**	**0,0064**	0,0703	0,4738	0,0746	**0,0074**	**0,0315**	**0,0269**
**SAL vs. DIL**	>0,9999						>0,9999	>0,9999	>0,9999	>0,9999	>0,9999
**SAL vs. 2H**	**0,0334**	0,1797	0,5075	0,1188	**0,0069**	**0,0406**	0,7252	>0,9999	0,3786	0,1323	0,0955
**EX1 vs. EX4**	>0,9999						>0,9999	>0,9999	>0,9999	>0,9999	>0,9999
**EX1 vs. DIL**	>0,9999	>0,9999	>0,9999	>0,9999	>0,9999	>0,9999	>0,9999	0,3276	0,3337	0,0784	0,1152
**EX1 vs. 2H**	0,0803	>0,9999	0,9165	>0,9999	>0,9999	>0,9999	>0,9999	>0,9999	>0,9999	>0,9999	>0,9999
**EX4 vs. DIL**	0,0628						>0,9999	**0,015**	**0,0096**	0,1115	0,1879
**EX4 vs. 2H**	>0,9999	>0,9999	>0,9999	>0,9999	>0,9999	>0,9999	>0,9999	0,3719	>0,9999	>0,9999	>0,9999
**DIL vs. 2H**	0,095						>0,9999	>0,9999	0,453	0,3911	0,5235

Figure 4	*TUNEL*						
**Analysis**	**Overall**	**CTX**	**PYR**	**HIP**	**STR**	**THL**	**EXC**
**Kruskal-Wallis test *P* value**	**<0,0001**	**<0,0001**	**<0,0001**	**0,0027**	**<0,0001**	**<0,0001**	**<0,0001**
**Dunn's multiple comparisons test**	*P* Value	*P* Value	*P* Value	*P* Value	*P* Value	*P* Value	*P* Value
**SAL vs. EX4**	**0,0002**	**<0,0001**	**0,0001**	**0,0253**	**<0,0001**	**<0,0001**	**<0,0001**
**SAL vs. 2H**	**<0,0001**	**<0,0001**	**0,0002**	**0,0037**	**<0,0001**	**0,001**	**<0,0001**
**EX4 vs. 2H**	>0,9999	>0,9999	>0,9999	>0,9999	>0,9999	>0,9999	>0,9999

Figure 5	*Microglial activation*

Analysis	Overall	CTX	PYR	HIP	STR	THL	EXC
**Kruskal-Wallis test *P* value**	**<0,0001**	**<0,0001**	**<0,0001**	**<0,0001**	**<0,0001**	**<0,0001**	**<0,0001**
**Dunn's multiple comparisons test**	*P* Value	*P* Value	*P* Value	*P* Value	*P* Value	*P* Value	*P* Value
**SAL vs. EX4**	**0,0006**	**<0,0001**	**0,0002**	**<0,0001**	**<0,0001**	**<0,0001**	**<0,0001**
**SAL vs. 2H**	**<0,0001**	**0,0024**	**0,0011**	**0,0005**	**0,0001**	**<0,0001**	**0,0006**
**EX4 vs. 2H**	>0,9999	0,6218	>0,9999	0,9508	>0,9999	>0,9999	0,988

**Figure 5**	***Astrogliosis***						
**Analysis**	**Overall**	**CTX**	**PYR**	**HIP**	**STR**	**THL**	**EXC**
**Kruskal-Wallis test *P* value**	**0,002**	**0,0072**	**0,031**	**0,0001**	**0,0127**	0,205	**<0,0001**
**Dunn's multiple comparisons test**	*P* Value	*P* Value	*P* Value	*P* Value	*P* Value	*P* Value	*P* Value
**SAL vs. EX4**	**0,0038**	**0,0115**	**0,0551**	**0,0006**	**0,0279**	0,248	**0,0004**
**SAL vs. 2H**	**0,0155**	**0,0416**	0,0944	**0,0011**	**0,0412**	0,7109	**0,0005**
**EX4 vs. 2H**	>0,9999	>0,9999	>0,9999	>0,9999	>0,9999	>0,9999	>0,9999

**Figure 5**	***Endothelial activation***						
**Analysis**	**Overall**	**CTX**	**PYR**	**HIP**	**STR**	**THL**	**EXC**
**Kruskal-Wallis test *P* value**	**0,0037**	**<0,0001**	0,1101	**0,0379**	**0,0106**	**0,0138**	**0,0038**
**Dunn's multiple comparisons test**	*P* Value	*P* Value	*P* Value	*P* Value	*P* Value	*P* Value	*P* Value
**SAL vs. EX4**	**0,005**	**<0,0001**	0,1702	**0,0443**	**0,0136**	**0,0171**	**0,0057**
**SAL vs. 2H**	**0,0356**	**0,0004**	0,2727	0,192	0,0722	0,0904	**0,03**
**EX4 vs. 2H**	>0,9999	>0,9999	>0,9999	>0,9999	>0,9999	>0,9999	>0,9999

**Figure 6**	***Blood analysis***

Analysis	Total WBC	Neutrophils	Lymphocytes	Monocytes	Eosinophils	Basophils	HCT	Platelets	RBC	Haemoglobin	MCV
**Kruskal-Wallis test *P* value**	0,431	0,2456	0,1464	0,7529	0,6154	>0,9999	0,5148	0,4632	0,555	0,0771	0,242
**Dunn's multiple comparisons test**	*P* Value	*P* Value	*P* Value	*P* Value	*P* Value	*P* Value	*P* Value	*P* Value	*P* Value	*P* Value	*P* Value
**Naïve vs. SAL**	>0,9999	0,3957	>0,9999	>0,9999	0,6072	>0,9999	>0,9999	>0,9999	>0,9999	>0,9999	0,3919
**Naïve vs. EX4**	0,9152	0,3957	0,1524	>0,9999	0,5371	>0,9999	0,6962	>0,9999	0,1207	0,0876	>0,9999
**SAL vs. EX4**	0,6354	>0,9999	0,8175	>0,9999	>0,9999	>0,9999	>0,9999	0,6764	0,1473	0,4487	0,3833

**Figure 6**	***Naïve body weights***				
**Analysis**	**0h**	**12h**	**24h**	**36h**	**48h**
**Kruskal-Wallis test *P* value**	0,8507	0,062	**0,0147**	**0,0145**	**0,0137**
**Dunn's multiple comparisons test**	*P* Value	*P* Value	*P* Value	*P* Value	*P* Value
**Naïve vs. SAL**	>0,9999	>0,9999	>0,9999	>0,9999	>0,9999
**Naïve vs. EX4**	>0,9999	0,1832	**0,0552**	**0,0558**	0,0623
**SAL vs. EX4**	>0,9999	0,1022	**0,0552**	**0,0558**	0,0677

**Figure 6**	***Glucose***
**Analysis**	
**Kruskal-Wallis test *P* value**	0,5013
**Dunn's multiple comparisons test**	*P* Value
**Naïve vs. 0.5h**	>0,9999
**Naïve vs. 1h**	>0,9999
**Naïve vs. 2h**	>0,9999
**Naïve vs. 4h**	>0,9999
**0.5h vs. 1h**	>0,9999

**0.5h vs. 2h**	>0,9999
**0.5h vs. 4h**	>0,9999
**1h vs. 2h**	>0,9999
**1h vs. 4h**	>0,9999
**2h vs. 4h**	>0,9999

**Figure 7**	***CD68 immunoreactivity***						
**Analysis**	**Brain**	**Heart**	**Pancreas**	**Spleen**	**Liver**	**Lung**	**Kidney**
**Kruskal-Wallis test *P* value**	0,323	0,9671	0,1405	0,0657	0,313	0,0871	0,8393
**Dunn's multiple comparisons test**	*P* Value	*P* Value	*P* Value	*P* Value	*P* Value	*P* Value	*P* Value
**Naïve vs. SAL**	0,4789	>0,9999	0,7303	0,0662	0,4233	0,3419	>0,9999
**Naïve vs. EX4**	>0,9999	>0,9999	0,1418	0,4693	>0,9999	0,1006	>0,9999
**SAL vs. EX4**	0,6898	>0,9999	>0,9999	>0,9999	0,7587	>0,9999	>0,9999

**Figure 8**	***Score***	***NT/HT body weight***				***Volume***					
**Analysis**		**P10**	**P12**	**P14**	**P17**	**Treatment**	**L1**	**L2**	**L3**	**L4**	**L5**
**Kruskal-Wallis test *P* value**	**<0,0001**	0,1487	0,1487	**0,0107**	**0,0497**	**<0,0001**	**<0,0001**	**<0,0001**	**<0,0001**	**<0,0001**	**<0,0001**
**Dunn's multiple comparisons test**	*P* Value	*P* Value	*P* Value	*P* Value	*P* Value	*P* Value	*P* Value	*P* Value	*P* Value	*P* Value	*P* Value
**NT SAL vs. NT EX4**	**0,0004**	>0,9999	>0,9999	>0,9999	0,9782	**0,0003**	**0,0067**	**0,0019**	**0,0112**	**0,0016**	**0,0006**
**NT SAL vs. HT SAL**	**0,0171**	>0,9999	>0,9999	0,0785	0,1606	**0,0115**	0,1246	**0,0466**	**0,0287**	**0,0527**	**0,0206**
**NT SAL vs. HT EX4**	**<0,0001**	>0,9999	0,4885	0,317	0,0691	**<0,0001**	**<0,0001**	**<0,0001**	**<0,0001**	**<0,0001**	**<0,0001**
**NT EX4 vs. HT SAL**	>0,9999	>0,9999	0,9644	0,3984	>0,9999	>0,9999	>0,9999	>0,9999	>0,9999	>0,9999	>0,9999
**NT EX4 vs. HT EX4**	0,672	>0,9999	0,2899	0,1911	>0,9999	>0,9999	0.1413	0,6568	0,8593	>0,9999	>0,9999
**HT SAL vs. HT EX4**	0,0532	>0,9999	>0,9999	>0,9999	>0,9999	0,21	**0,0093**	0,0541	0,2763	0,2405	0,9044

**Suppl. Figure 4**	***cAMP***
**Analysis**	

**Kruskal-Wallis test *P* value**	**0,0017**
**Dunn's multiple comparisons test**	*P* Value
**SAL/2h vs. 2h**	**0,0021**
**SAL/2h vs. 4h**	**0,0281**
**SAL/2h vs. 8h**	0,8308
**SAL/2h vs. 12h**	>0,9999
**2h vs. 4h**	>0,9999
**2h vs. 8h**	0,486
**2h vs. 12h**	0,1018
**4h vs. 8h**	>0,9999
**4h vs. 12h**	0,6394
**8h vs. 12h**	>0,9999

Bold *p* values indicate significance at $p<0.05$
Abbreviations: 2H - exendin 4, 4 high-doses 2h delay; CTX – isocortex; DIL – exendin 4, 4 low-doses; EX1 – exendin 4, 1 high-dose; EX4 – exendin 4, 4 high-doses; EXC – external capsule; HIP – hippocampus; HT – hypothermia; NT – normothermia; PYR – pyriform cortex; THL – thalamus; SAL – saline; STR – striatum.

Table 3. Hypoxia-ischemia experimental animals listed by sex

	P7 late preterm HI					*P10 term HI*			
	**Saline**	**1 high-dose**	**4 high-doses**	**2h delay**	**4 low-doses**	**NT SAL**	**NT EX4**	**HT SAL**	**HT EX4**
**1**	M	M	M	F	F	F	M	F	M
**2**	F	M	M	M	F	F	M	M	F
**3**	M	F	F	F	F	M	M	M	F
**4**	F	F	M	M	M	F	M	M	F
**5**	M	F	F	M	M	F	M	F	M
**6**	M	F	F	M	M	F	F	M	F
**7**	M	M	F	M	M	M	M	M	F
**8**	F	M	M	F	M	F	F	M	M
**9**	F	M	M	F	F	F	F	F	M
**10**	M	M	M	F	F	F	M	F	M
**11**	M	F	F	M	M	M	M	M	F
**12**	F	M	F	F	F	F	F	M	F
**13**	F	M	F	M	M	F	F	M	M
**14**	M	F	M	F	F	M	M	F	M
**15**						M	M	F	M
**16**						F	M	M	M
**17**						M	F	M	F
**18**						F	F	M	F
**19**						F	F	M	F
**20**						M	F	F	F
**21**						M	F	F	F
**22**						M	F	F	F
**23**						M	M	F	M
**24**						F	F	F	M
**25**							F	M	M

Abbreviations: EX4 – exendin-4; F – female; HI – hypoxia-ischemia; HT – hypothermia; M – male; NT – normothermia; P – postnatal day; SAL – saline.
